# Integrating concept of pharmacophore with graph neural networks for chemical property prediction and interpretation

**DOI:** 10.1186/s13321-022-00634-3

**Published:** 2022-08-04

**Authors:** Yue Kong, Xiaoman Zhao, Ruizi Liu, Zhenwu Yang, Hongyan Yin, Bowen Zhao, Jinling Wang, Bingjie Qin, Aixia Yan

**Affiliations:** 1 State Key Laboratory of Chemical Resource Engineering, Department of Pharmaceutical Engineering, Beijing University of Chemical Technology, 15 BeiSanHuan East Road, P. O. Box 53, Beijing, 100029 People’s Republic of China; 2 Hyper-Dimension Insight Pharmaceuticals Ltd. Room 511, Block A, No. 2C, DongSanHuan North Road, Beijing, People’s Republic of China

**Keywords:** Graph neural networks (GNNs), Pharmacophore, Reduced graph (RG), Hierarchical pooling

## Abstract

Recently, graph neural networks (GNNs) have revolutionized the field of chemical property prediction and achieved state-of-the-art results on benchmark data sets. Compared with the traditional descriptor- and fingerprint-based QSAR models, GNNs can learn task related representations, which completely gets rid of the rules defined by experts. However, due to the lack of useful prior knowledge, the prediction performance and interpretability of the GNNs may be affected. In this study, we introduced a new GNN model called RG-MPNN for chemical property prediction that integrated pharmacophore information hierarchically into message-passing neural network (MPNN) architecture, specifically, in the way of pharmacophore-based reduced-graph (RG) pooling. RG-MPNN absorbed not only the information of atoms and bonds from the atom-level message-passing phase, but also the information of pharmacophores from the RG-level message-passing phase. Our experimental results on eleven benchmark and ten kinase data sets showed that our model consistently matched or outperformed other existing GNN models. Furthermore, we demonstrated that applying pharmacophore-based RG pooling to MPNN architecture can generally help GNN models improve the predictive power. The cluster analysis of RG-MPNN representations and the importance analysis of pharmacophore nodes will help chemists gain insights for hit discovery and lead optimization.

**Graphical Abstract:**

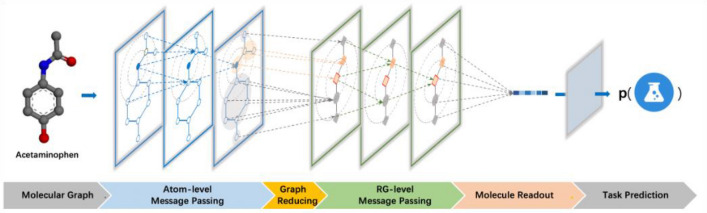

**Supplementary Information:**

The online version contains supplementary material available at 10.1186/s13321-022-00634-3.

## Introduction

With the accumulation of large-scale chemical and biological data, the improvement of computing power, and especially the major breakthroughs that deep neural networks (DNNs) having made in many fields such as image recognition [1] and natural language processing [2], there has been a surge of interest in developing DNNs for drug discovery in recent years [3–7]. This trend is especially reflected in the development of a variety of DNNs for chemical property prediction [8–10]. In the field of drug design, these models correspond to the quantitative structure–activity (property) relationship (QSAR/QSPR) models [11], belonging to the category of ligand-based drug design (LBDD) method [12, 13]. This method is not limited to the availability of the 3D structure of the target of interest but fits models or finds patterns from the collected ligand data, which is commonly used for large-scale virtual screening, chemical property evaluation and molecular structure optimization.

Before the rise of DNNs, there have been extensive QSAR models developed for drug discovery, mainly using traditional machine learning (ML) approaches, such as support vector machines (SVMs) [14, 15], Naïve Bayes (NB) [16, 17], artificial neural network (ANN) [18, 19], random forest (RF) [20] etc. Although many reports claimed that the performance of many DNN models did not meet researchers’ expectation that the prediction accuracy is far beyond traditional machine learning [21, 22], it is largely because the amount of data is too small to give full play to advantages of DNNs. This advantage is bound to be brought into play as the amount of chemical and biological data gradually accumulates. Another point that motivates researchers to keep great enthusiasm for the development of DNNs is their ability of effective representation extraction. Instead of using expert features and performing feature engineering like traditional machine learning models, which is complex and time-consuming, not easy to reproduce, and limited by expert feature definition, DNNs can extract useful representations from task data for the sake of the end-to-end fashion. And this task-learned representations can be used for analogues searching in virtual screening campaigns and gaining insights of relationship between chemical structure and properties to guide molecular optimization. However, to achieve accurate predictions and extract useful representations at the same time depends on well-designed DNN architecture, which is challenging but urgent to be paid efforts to.

Given that the framework of DNN is quite flexible, there have been a variety of DNN models published for drug discovery. Most of these models can be mainly divided into two types: one is to use the RNN (recurrent neural network) or Transformer [23] frameworks to operate the molecular SMILES as strings [24, 25], the other one is to use the GNN (graph neural network) framework to operate molecules as graphs [26, 27]. Although systematic performance comparison results for these two methods are rare, GNN is more popular than RNN in chemical property prediction in recent years. This may be because the form of graph representation is closer to the intrinsic properties of the molecular structure, thus what the model learns from the graph is more able to reflect the properties of the molecule. On the other hand, the SMILES string guides the model to learn a lot of SMILES grammar rules, such as parentheses representing branched chains which are irrelevant to molecular structure. Recently, as a general GNN architecture, the message-passing neural network (MPNN) [28] has been proposed, consisting of a message-passing phase and a readout (or called pooling) phase. Researchers have developed many models based on MPNN architecture to predict chemical properties and extract task-learned representations, such as MPNN model (note it refers a specific model instead the previously mentioned MPNN architecture), D-MPNN (Directed MPNN) [29], AttentiveFP [30], R-GCN (Relational Graph Convolutional Networks) [31] and GSN (Graph Substructure Networks) [32].

However, most of the MPNN architectures only absorb node information (such as atom type, formal charge) and edge information (such as bond type, stereo type) as the original information of a molecular graph, but do not make full use of prior chemical knowledge, such as the information from pharmacophores [33], which have been widely used in drug design and discovery. Of note, there have been many successful cases that prove that the pharmacophore rules can be well combined with molecular graph, and one representative method is the pharmacophore-based graph reduction [34–36]. Based on pharmacophore rules, the reduced graphs (RGs) provide simplified representations of chemical structures by collapsing atom groups into pharmacophores while maintaining the topological properties of the original molecular structures. This drives us to think about whether embedding information of pharmacophores into MPNN architecture under graph reduction scheme will help improve the accuracy and reliability of the model and to enrich the information contained in the task-learned representation.

Another limitation of current MPNN architectures for chemical property prediction is that they ignore any hierarchical structure and information that might exist in the molecular graph, which will hinder the models from effectively extracting the information in the graph. On the other hand, the global pooling such as the maximizing pooling, average pooling, and pooling with attention mechanisms [30], has been adopted as the standard readout phase for a model with the MPNN architecture, leading to a “flat” nature. More recently, hierarchical pooling has attracted research attention, for instance, Diffpool [37] uses a learned distribution matrix to collapse atom groups. However, these current hierarchical structures still do not make full use of prior chemical knowledge. To date, there has been no hierarchical pooling method leverages knowledge of pharmacophore to design GNN models, specifically, the ones with the MPNN architecture.

**Fig. 1:**
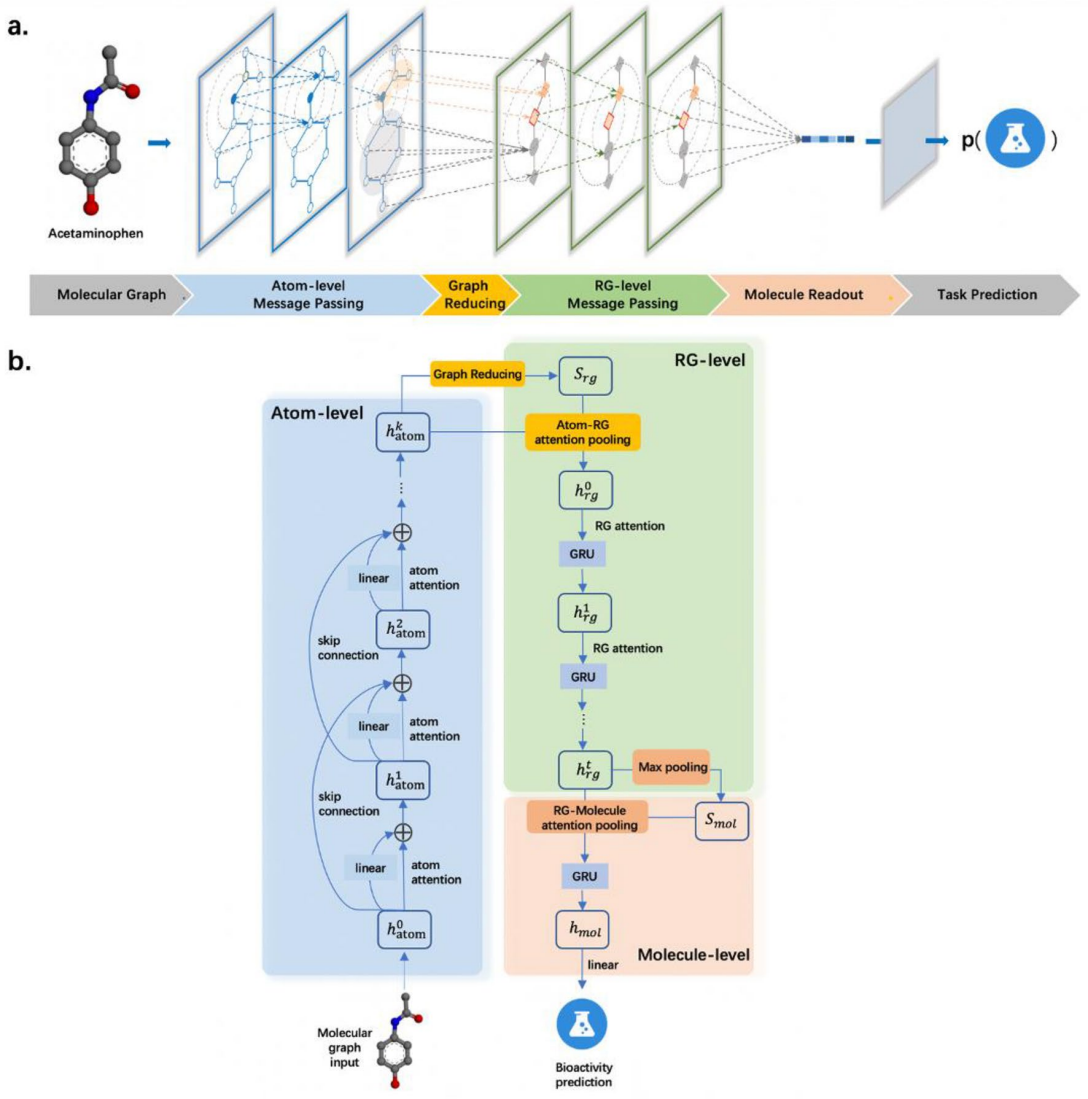
Illustration of our proposed RG-MPNN. **a** General architecture of RG-MPNN. Taking Acetaminophen as an example, there are four phases that a molecule goes through from the molecular graph to the task prediction. **b** Specific architectural details of each phase of RG-MPNN

In this work, we proposed a new GNN model, RG-MPNN, for chemical property prediction. The core idea of RG-MPNN was to integrate pharmacophore information hierarchically into MPNN architecture, specifically, in the way of pharmacophore-based RG pooling. As illustrated in Fig. 1, the RG-MPNN absorbed not only the information of atoms and bonds from the atom-level message-passing phase, but also the information of pharmacophores from the RG-level message-passing phase. Our models achieved state-of-the-art prediction performance and these results were also transferred to data sets of ten popular kinases. Furthermore, the cluster analysis of the task-learned representation of RG-MPNN showed that the representation can be used to identify molecules with similar activities but different scaffolds in the context of virtual screening and lead optimization.

## Methods

### Data sets

#### Benchmark datasets

To compare the performance of the RG-MPNN with those of other GNN models, we tested our models on eleven benchmark datasets from MoleculeNet [38]. Among MoleculeNet, the physical chemistry, bioactivity and physiology data sets except for the PDBbind data sets were tested in this work. Three of the eleven datasets were used for regression tasks and eight for classification tasks. More details about the datasets from MoleculeNet can be found on the website https://moleculenet.org/datasets-1.

#### Kinase datasets and some in-house datasets

The core idea of our model RG-MPNN is to integrate information of pharmacophores which are regarded as abstract features of molecules for molecular recognition of a ligand by a biological target. Therefore, in theory, our model is more suitable for the task of predicting molecular bioactivity towards targets of interest. To systematically test the prediction performance of various algorithms on the bioactivity datasets, we collected inhibitors of ten kinase targets (see Table 1). The principle of kinase target selection was to cover each kinase family as much as possible and select the targets of great prospects for drug development. All these datasets were derived from ChEMBL [39]. After a series of operations such as data deduplication, salt removal, and electrical neutrality, ten kinase data sets were ready for classification task. We used 1000 nM as a threshold to distinguish active and inactive molecules, resulting in the numbers of molecules ranging from 807 to 8800 and the ratios of positive and negative samples ranging from 0.19 to 0.82. In addition to the kinase datasets, we also used some datasets (HCV NS3, PLA2, HIV protease, and Tyrosinase) published by our lab as a reference. For detailed dataset descriptions, see Additional file 1: Table S1.

**Table 1:** Basic information of kinase datasets used in this work

Kinase family	Kinase full name^a^	Short name	Total^b^	Active	Inactive	Ratio^c^
TK	Epidermal growth factor receptor erbB1	EGFR	8718	4514	4286	0.52
TKL	Serine/threonine-protein kinase B-raf	BRAF	4669	3629	1132	0.78
CAMK	Serine/threonine-protein kinase PIM1	PIM1	4437	3322	1185	0.75
Atypical	Serine/threonine-protein kinase mTOR	mTOR	3924	3390	745	0.86
AGC	Serine/threonine-protein kinase AKT	AKT1	3904	2175	1792	0.56
Other	Serine/threonine-protein kinase Aurora-A	AURKA	3854	2286	1596	0.59
TK	Tyrosine-protein kinase BTK	BTK	2560	1894	746	0.74
CMGC	Cyclin-dependent kinase 2	CDK2	2531	1302	1301	0.51
STE	Mitogen-activated protein kinase kinase kinase kinase 2	MAP4K2	891	280	617	0.31
CK1	Casein kinase I alpha	CK1	801	154	653	0.19

^a^preferred name listed on ChEMBL website

^b^number of molecules in total

^c^active/total ratio

### Molecular graph

In graph neural networks (GNNs), a molecule is regarded as a graph G = (V, E), where atom is regarded as node V and chemical bond is regarded as edge E. The nodes and edges are encoded according to the rules shown in the Additional file 1: Table S2 and Additional file 1: Table S3. For instance, node features include atom type, formal charge, etc., and edge features include bond type, stereo type, etc. These encoded features are the initial features of molecular graphs which are used as raw inputs to train GNN models. After training, we can get the final task prediction value, together with the task-learned graph representations that also can be called molecular fingerprints.

### Reduced graphs (RGs)

RGs provide simplified representations of chemical structures by collapsing atom groups into pharmacophore nodes while maintaining the topological properties of the original molecular structures. RGs have been mainly implemented to the varied applications of similarity searching, scaffold hopping, de novo design and structure–activity relationships extracting [34, 36, 40, 41].

By altering the rules used for collapsing atom groups, RGs provide flexible ways of generalizing pharmacophore node features. There is a research trend to collapse the atom groups into RGs through the pharmacophore rules and the resulting RGs can be regarded as topological pharmacophore [36, 40]. It is worth emphasizing that the pharmacophore rules need to be improved before applied to graph reduction. This is because each atom in RGs needs to be mapped to one or more pharmacophore nodes, while atoms that do not belong to any pharmacophore are not labeled according to classical pharmacophore rules.

**Fig. 2:**
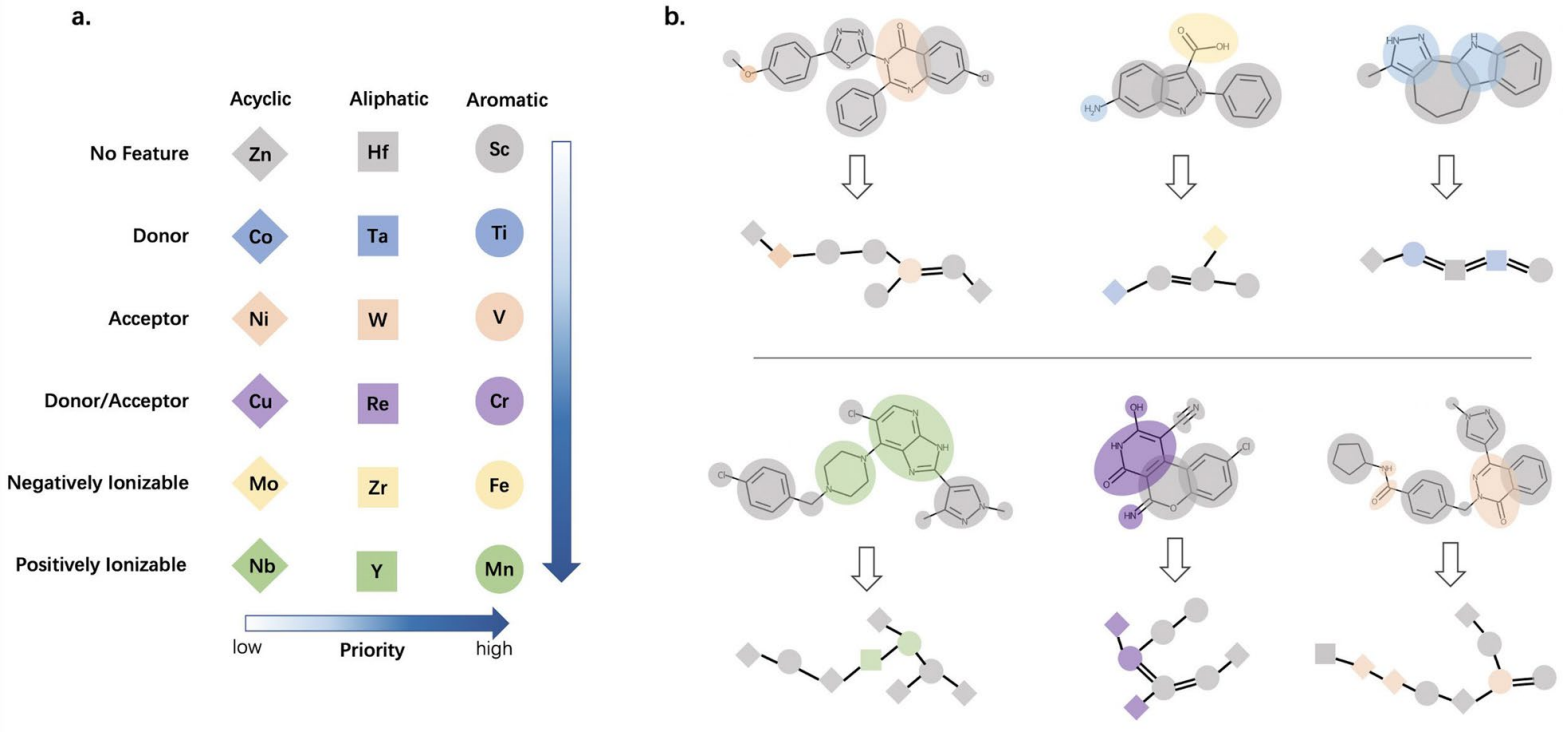
Scheme and examples of graph reduction. **a** The graph reduction scheme adopted in this work. The three ways of defining rings and six ways of defining features combine to eighteen types of reduced graphs. And prioritize at the ring and feature level. **b** Comparative examples of molecules and their reduced graphs

In this work, we adopted the graph reduction scheme developed by Harper [34], which defines 18 types of pharmacophore nodes as shown in Fig. 2a: three types about defining rings (aromatic ring, aliphatic ring, or acyclic) intersected with six types about defining features (positively ionizable, negatively ionizable, joint H-bond donor and acceptor, donor, acceptor, or no feature), and it should be noted that the items within the three ring types and the six feature types are listed in order of priority from high to low. See Additional file 1: Table S4 for the detailed rule descriptions of the six feature types. Figure 2b lists some comparative examples of molecules and their RGs. Readers can find more graph reduction schemes in literature [34, 35, 42].

### Message-passing neural network (MPNN)

MPNN is a general framework for supervised learning on graphs. Within its forward pass, there are two phases: a message-passing phase and a readout phase. Here we take an undirected graph $G$ as an example, within which the node (atom) features are represented as ${x}_{v}$ and the edge (bond) features as ${e}_{vw}$. In terms of the message-passing phase, the message function is defined as ${M}_{t}$, and the vertex update function is defined as ${U}_{t}$, where $t$ is the running time step. During message-passing process, the hidden state of each node ${h}_{v}^{t+1}$ can be updated based on message ${m}_{v}^{t+1}$ according to:

1 $$m_{v}^{t + 1} = \sum\nolimits_{w \in N\left( v \right)} {M_{t} \left( {h_{v}^{t} ,h_{w}^{t} ,e_{vw} } \right)}$$

2 $$h_{v}^{t + 1} = U_{t} \left( {h_{v}^{t} ,m_{v}^{t + 1} } \right)$$

where $N\left(v\right)$ is the set of neighbors of the node $v$ in $G$. In addition, ${h}_{v}^{0}$ is derived from the initial node features ${x}_{v}$ through some function.

In terms of the readout phase, it uses a readout function $R$ to make a task prediction for the whole graph according to:

3 $$\hat{y} = R\left( {\left\{ {h_{v}^{T} |v \in G} \right\}} \right)$$

where the output $\widehat{y}$ can be a scalar or a vector, depending on whether it is used for single task prediction or multi-task predictions.

During training process, taking the molecular graphs as inputs, the model predicts the properties of each molecule. The loss is computed based on the predicted properties and the true ones, then of which the gradient is backpropagated through the readout phase and the message-passing phase.

### Applying reduced-graph to MPNN architecture

Adding reduced-graph pooling to message-passing neural network architecture was proposed in this work, which results in four phases: a message-passing phase at atom level, a graph reducing phase, a message-passing phase at RG level and a molecule readout phase. These four phases correspond to the schematic in Fig. 1a and Additional file 1: Table S5. In short, compared with common MPNN architecture, the proposed architecture has one more graph-reducing phase and one message-passing phase at RG level. The MPNN architecture with RG pooling works as follows.

#### Atom-level message-passing

During the atom-level message-passing phase, the operation of MPNN architecture with RG pooling is very similar to the message-passing phase of typical MPNNs, with one difference that $e$ is not directly considered in the message function ${M}_{k}$ since ${h}_{{atom}^{^{\prime}}}$ is derived from $cat({x}_{ {atom}^{^{\prime}}},{e}_{atom-{atom}^{^{\prime}}})$ by linear transformation. This phase runs for $K$ time steps. The hidden state of each atom ${h}_{atom}^{k+1}$ can be updated based on message ${m}_{atom}^{k+1}$ according to:

4 $$m_{atom}^{k + 1} = \sum\nolimits_{{atom^{\prime} \in N\left( {atom} \right)}} {M_{k} \left( {h_{atom}^{k} ,h_{atom^{\prime}}^{k} } \right)}$$

5 $$h_{atom}^{k + 1} = U_{k} \left( {h_{atom}^{k} ,m_{atom}^{k + 1} } \right)$$

#### Graph reducing

During this phase, the whole graph $G$ is operated by the function $Reduce$ which maps each atom to one or more pharmacophore nodes with the rules we have mentioned in the method part of reduced graphs, resulting in a reduced graph $RG$. Then, we define ${RG= (V}^{^{\prime}}, {E}^{^{\prime}})$, where ${V}^{^{\prime}}$ represents the pharmacophore node, which is one of the 18 predefined pharmacophore nodes, and ${E}^{^{\prime}}$ represents the edge between pharmacophore nodes, which is equal to one plus to the number of chemical bonds shared between two adjacent pharmacophore nodes. The hidden state of initial pharmacophore node ${h}_{rg}^{0}$ according to:

6 $$h_{ra}^{o} = {\text{Re}} duce\left( {\left\{ {h_{atom}^{k} |atom \in V^{\prime}} \right\}} \right)$$

#### RG-level message-passing

This phase runs for $T$ time steps and the hidden state of each pharmacophore node ${h}_{rg}^{t+1}$ can be updated based on message ${m}_{rg}^{t+1}$ according to:

7 $$m_{rg}^{t + 1} = \sum\nolimits_{{rg^{\prime} \in N\left( {rg} \right)}} {M_{t} \left( {h_{rg}^{t} ,h_{rg^{\prime}}^{t} } \right)}$$

8 $$h_{rg}^{t + 1} = U_{t} \left( {h_{rg}^{t} ,m_{rg}^{t + 1} } \right)$$

#### Molecule readout

During this phase, the molecule embedding ${h}_{mol}$, also as the task-learned representation of molecular graph, is achieved by a readout function $R$ based on the hidden states ${h}_{rg}^{T}$ within $RG$:

9 $$h_{mol} = R\left( {\left\{ {h_{rg}^{T} |V^{\prime} \in RG} \right\}} \right)$$

then the prediction of molecular property is achieved through MLP layers:

10 $$\hat{y} = MLP\left( {h_{mol} } \right)$$

where the output $\widehat{y}$ can be a scalar or a vector same as that in the MPNN process, depending on whether it is used for single task prediction or multi-task predictions.

Theoretically, the MPNN architecture with RG pooling proposed in this paper can be applied to any model under the MPNN architecture, that is, before readout of the whole molecule, the graph reducing and message-passing at RG level can added, and the latter operation is optional.

### RG-MPNN

Under the MPNN architecture with RG pooling, we proposed a model called RG-MPNN (short for reduced-graph message-passing neural network), which was designed by adding RG pooling based on the residual message-passing neural network (shorted as ResMPNN). As shown in Fig. 1b, the RG-MPNN follows four processes mentioned above in turn: a message-passing phase at atom level, a graph reducing phase, a message-passing phase at RG level and a molecule readout phase.

At atom level, RG-MPNN shares the same message-passing phase and the update phase as the base model—ResMPNN. Within the message-passing phase, when gathering messages from neighbor atoms, our model adopts the attention mechanism, which was proposed by Velickovic and Bengio et al. in constructing GAT [43] model. The core idea of the attention mechanism is to receive messages from neighbors according to a certain weight that is calculated based on the feature vectors of the center atom and its neighbor atom. This mechanism is in line with our basic chemical understanding, that is, each atom is influenced by its neighbor atom with different degrees, which may lie in factors such as the strength of the electrostatic attraction, the shift of the electron cloud, etc. Moreover, in the update process, the new hidden state of atom ${h}_{atom}^{k}$ is obtained by adding attention message and residuals. It is worth emphasizing that there are $k-1$ residual items based on skip-connection mechanism, the linearly transformed values of the previous hidden states (*e.g.* ${h}_{atom}^{1}$ and ${h}_{atom}^{2}$ when $k=3$), since the skip-connection residual can effectively avoid the problem of gradient disappearance during process of the network training.

The graph reducing process can be regarded as a pharmacophore-based graph reduction along with a one-step message-passing. Firstly, the graph $G$ is reduced into $RG$ (reduced graph) according to the previously defined pharmacophore-based RG pooling rules, and the sum of vectors of child nodes inside one pharmacophore node is regarded as the initial state ${S}_{rg}$. Then it comes to the message-gathering step in MPNN architecture. Each pharmacophore node receives the messages from their child nodes through their attention weights. This is consistent with the chemical intuition that each atom contributes differently to its pharmacophore. Finally, the pharmacophore nodes are updated through a GRU (gated recurrent unit) [44], with the expectation that the network can weigh the initial state ${S}_{rg}$ and the messages passed over.

During the message-passing phase at RG level, the operation is similar to the MPNN step in the graph reducing process, that is, the attention mechanism is applied to gather messages, and then the GRU is used to update the nodes.

The implementation of the molecule readout is very similar to that of graph reducing process since the readout operation can be regarded as a special case of graph reducing, that it, all child nodes belong to one pharmacophore node, with the sum of vectors of these child nodes as the initial state at molecule level ${S}_{mol}$.

### Model evaluation

In this work, we used two methods to split each dataset into a training set, a validation set and a test set. The first was to split randomly according to the ratio of 8: 1: 1. Noted that in each round of comparing the performance of algorithms, the random seed was kept the same to eliminate the impact of different dataset divisions. Each dataset was randomly split five times, and we built a model based on each split dataset, so a total of five models were built for each dataset. The second is scaffold splitting. The core idea of scaffold splitting is to put molecules with different scaffolds into different sets to evaluate the prediction ability on new scaffolds that not encountered during training. Each dataset was also randomly split five times similar to the method mentioned above, under the premise of ensuring that molecules with the same scaffold are divided into the training set, validation set or test set at the same time. Note that the error bars on all plots show the standard error of the mean across five runs, where standard error is defined as the standard deviation divided by the square root of five (the number of runs).

For the benchmark data sets, we used RMSE (root mean square error) to evaluate regression tasks, and AUC (area under curve) to evaluate classification tasks, to be consistent with other models on benchmark evaluation. For kinase data sets, two indicators were used to evaluate the model—AUC and MCC (Matthews correlation coefficient), as the two are not sensitive to data imbalance [45]. In different scenarios, the best model can be selected according to different indicators. The AUC indicator is suitable to select models in the scenarios where the correct sorting is counted such as shortlisting compounds for bioactivity testing in virtual screening, since it measures the ability of model to rank positive samples before negative ones. While the MCC indicator is suitable for the models used in the scenarios where the correct classification is counted such as evaluating whether the molecule is active or toxic.

### Model training and hyper-parameter search

Pytorch [46], a deep-learning framework, was used for developing all parts of the RG-MPNN, RDKit (v.2018.09.2) [47] for processing molecules and Pytorch Geometric [48] for transforming a molecule into a graph. MSELoss and CrossEntropyLoss were used as loss functions for regression and classification tasks, respectively, whereas Adam [49] was used for gradient descent optimization. For each dataset, we adopted random hyper-parameter search by using the Python package NNI (https://github.com/microsoft/nni). The following six hyper-parameter together with their scope of the choice, base_lr (base L2 weight decay): [1e-3, 1e-4], k (times of message -passing layers at atom level): [2–5], t (times of message -passing layers at rg level): [1–3], batch: [16, 32], fingerprint dimension: [64, 128, 256, 512], dropout rate: [0, 0.1, 0.2, 0.3, 0.4, 0.5]. Combined with early stop strategy, the best parameters were selected based on the performance of the validation dataset. This work used the same strategy to do hyper-parameter search for MPNN model and AttentiveFP model. In addition, Additional file 1: Table S6 lists the number of parameters and the average running time of the main models (RG-MPNN, AttentiveFP and MPNN models) in this work.

## Results and discussion

### Model performance on benchmark data sets

To compare the performance of RG-MPNN with other existing GNNs, we used the benchmark data sets to test these models. Considering the reported performance and code availability of the models, we selected MPNN [28] and AttentiveFP [30] for comparison. The former is the classic MPNN model as the baseline, and the latter is the model with superior performance reported in the recent period. We list the model performance of some commonly used GNN models and machine learning models on the benchmark datasets, including AttentiveFP, MPNN, GC, Weave, D-MPNN, SVM, XGBoost and RF models. See Additional file 1: Table S7 for model performance and literature reference. In this work, we reproduced these models, trained, and tested them on datasets locally. The reason why we did not directly compare the performance of the RG-MPNN with that of models listed in Additional file 1: Table S7 for these reasons: (1) we used different data sets (our model can’t deal with the case that the molecule belongs to a single pharmacophore, and thus it is impossible to transfer information at the reduced graph level); (2) cannot obtain the same training set reported in the original literature; (3) there are some randomness when optimizing and training model parameters. Moreover, comparing the effects of GNN models and machine learning models is not the focus of this work, so we don’t reproduce these machine learning models. Readers can refer to the results of related literature for more information [21].

Table 2 and Additional file 1: Figure S1 summarize the performance on the benchmark data sets covering a variety of molecular bioactivities, toxicities, and physical chemistry properties. On the three regression tasks (ESOL, FreeSolv and Lipophilicity), it performed best in the comparison of locally reproduced models (see numbers in bold in Table 2). For classification tasks, our model performed best on six of the eight ones (HIV, MUV, BACE, Tox21, ToxCast and ClinTox, see numbers in bold in Table 2), which indicates that our model performed well for bioactivity and tox prediction tasks. It is worth emphasizing that toxicity data sets are all multi-task classification tasks, so this indicates the potential of our models in multi-task prediction, and more extensive experiments are worthy to test this hypothesis.

**Table 2:** Model performance on benchmark datasets

Category	Dataset	# Compounds^a^	Task type	# Tasks	Metrics	AttentiveFP	MPNN	RG-MPNN (our model)
Physical chemistry	ESOL	1030	Regression	1	RMSE	0.650 ± 0.123	0.853 ± 0.057	**0.605 ± 0.037**
	FreeSolv	566	Regression	1	RMSE	1.162 ± 0.180	1.255 ± 0.229	**0.939 ± 0.067**
	Lipophilicity	4085	Regression	1	RMSE	0.627 ± 0.055	0.662 ± 0.019	**0.579 ± 0.020**
Bioactivity	MUV	91,470	Classification	17	ROC-AUC	0.772 ± 0.031	0.740 ± 0.012	**0.819 ± 0.011**
	HIV	38,686	Classification	1	ROC-AUC	0.815 ± 0.022	0.803 ± 0.015	**0.824 ± 0.019**
	BACE	1419	Classification	1	ROC-AUC	0.868 ± 0.024	0.846 ± 0.026	**0.889 ± 0.018**
Physiology or toxicity	BBBP	1928	Classification	1	ROC-AUC	**0.888 ± 0.025**	0.824 ± 0.038	0.879 ± 0.035
	Tox21	7372	Classification	12	ROC-AUC	0.852 ± 0.025	0.836 ± 0.018	**0.873 ± 0.008**
	ToxCast	8058	Classification	617	ROC-AUC	0.860 ± 0.012	0.848 ± 0.008	**0.866 ± 0.009**
	SIDER	1270	Classification	27	ROC-AUC	**0.827 ± 0.008**	0.812 ± 0.012	0.825 ± 0.014
	ClinTox	1437	Classification	2	ROC-AUC	0.940 ± 0.029	0.941 ± 0.026	**0.965 ± 0.011**

Note that models with the best performance are in bold

^a^number of compounds used in this work

Overall, from the comparison of the models, our model RG-MPNN performed slightly better than AttentiveFP, and to a large extent better than the MPNN model, suggesting our model is a promising method to solve problems in drug discovery especially for bioactivity and tox prediction problems.

### Predicting target bioactivities

RG-MPNN integrates the concept of pharmacophore from drug discovery field into the graph neural network, aiming to improve the predicting ability for molecular bioactivity towards targets of interest. Under these circumstances, we constructed a series of kinase molecular activity data sets, aiming to test the model’s ability to predict molecular bioactivity on a larger scale. In addition to the data sets split randomly, we also trained and evaluated models on data sets split on scaffolds. This is because there has been research shown that the model trained on the data sets split based on scaffolds has better generalization ability in industry, given that this split can simulate the scene of the data set split by time periods in industry [38].

Table 3 lists the model performance under two indicators—MCC and AUC. Here, we only compare the models in terms of AUC. On randomly divided data sets, our model performed slightly better than AttentiveFP, achieving the best performances on seven out of ten, while AttentiveFP performed best on the other three data sets. In addition, both models mentioned above outperformed the MPNN model. The result is in line with our expectations because the pooling method is very important for task prediction. In theory, the hierarchical pooling of RG-MPNN and the attention pooling of AttentiveFP can extract representation or fingerprints more effectively than the average pooling in typical models with MPNN architecture.

**Table 3:** Model performance on kinase datasets

Dataset	Splitting	MPNN		AttentiveFP		RG-MPNN	
		MCC	AUC	MCC	AUC	MCC	AUC
EGFR	Random	0.699 ± 0.022	0.923 ± 0.009	0.729 ± 0.017	0.933 ± 0.010	**0.738 ± 0.029**	**0.942 ± 0.008**
	Scaffold	0.648 ± 0.03	0.901 ± 0.010	0.684 ± 0.029	0.908 ± 0.006	**0.706 ± 0.020**	**0.925 ± 0.007**
BRAF	Random	0.703 ± 0.052	0.916 ± 0.020	0.753 ± 0.035	**0.938 ± 0.006**	**0.774 ± 0.020**	0.915 ± 0.012
	Scaffold	0.614 ± 0.040	0.883 ± 0.006	**0.685 ± 0.018**	0.905 ± 0.008	0.677 ± 0.044	**0.915 ± 0.006**
PIM1	Random	0.691 ± 0.069	0.933 ± 0.029	0.741 ± 0.026	**0.957 ± 0.009**	**0.758 ± 0.029**	0.951 ± 0.013
	Scaffold	0.612 ± 0.050	0.881 ± 0.024	0.681 ± 0.021	0.921 ± 0.006	**0.710 ± 0.042**	**0.928 ± 0.007**
mTOR	Random	0.591 ± 0.037	0.888 ± 0.036	0.641 ± 0.033	**0.927 ± 0.010**	**0.674 ± 0.038**	0.921 ± 0.008
	Scaffold	0.408 ± 0.060	0.792 ± 0.033	**0.588 ± 0.022**	0.876 ± 0.011	0.574 ± 0.015	**0.886 ± 0.009**
AKT1	Random	0.669 ± 0.068	0.905 ± 0.035	0.751 ± 0.038	0.933 ± 0.011	**0.771 ± 0.031**	**0.941 ± 0.014**
	Scaffold	0.605 ± 0.041	0.883 ± 0.016	**0.657 ± 0.029**	**0.914 ± 0.010**	0.649 ± 0.023	0.910 ± 0.008
AURKA	Random	0.634 ± 0.061	0.892 ± 0.020	0.665 ± 0.021	0.909 ± 0.010	**0.690 ± 0.028**	**0.917 ± 0.007**
	Scaffold	0.471 ± 0.031	0.793 ± 0.012	0.475 ± 0.023	0.807 ± 0.008	**0.522 ± 0.037**	**0.836 ± 0.006**
BTK	Random	0.670 ± 0.065	0.915 ± 0.022	0.748 ± 0.083	0.947 ± 0.017	**0.759 ± 0.042**	**0.954 ± 0.017**
	Scaffold	0.545 ± 0.044	0.849 ± 0.021	0.626 ± 0.044	**0.902 ± 0.014**	**0.682 ± 0.031**	0.893 ± 0.015
CDK2	Random	0.567 ± 0.041	0.865 ± 0.019	0.624 ± 0.074	0.886 ± 0.021	**0.652 ± 0.050**	**0.902 ± 0.013**
	Scaffold	0.376 ± 0.052	0.752 ± 0.024	0.412 ± 0.026	0.773 ± 0.022	**0.495 ± 0.015**	**0.820 ± 0.008**
MAP4K2	Random	0.457 ± 0.131	0.792 ± 0.059	0.484 ± 0.123	0.813 ± 0.041	**0.540 ± 0.046**	**0.863 ± 0.025**
	Scaffold	0.174 ± 0.039	0.578 ± 0.037	0.277 ± 0.03	0.652 ± 0.033	**0.306 ± 0.045**	**0.706 ± 0.034**
CK1	Random	0.156 ± 0.162	0.673 ± 0.111	0.313 ± 0.128	0.751 ± 0.047	**0.433 ± 0.090**	**0.800 ± 0.065**
	Scaffold	− 0.020 ± 0.051	0.576 ± 0.029	0.159 ± 0.084	0.653 ± 0.011	**0.333 ± 0.107**	**0.687 ± 0.033**

Note that the best AUC and MCC for each kinase target are in bold

**Fig. 3:**
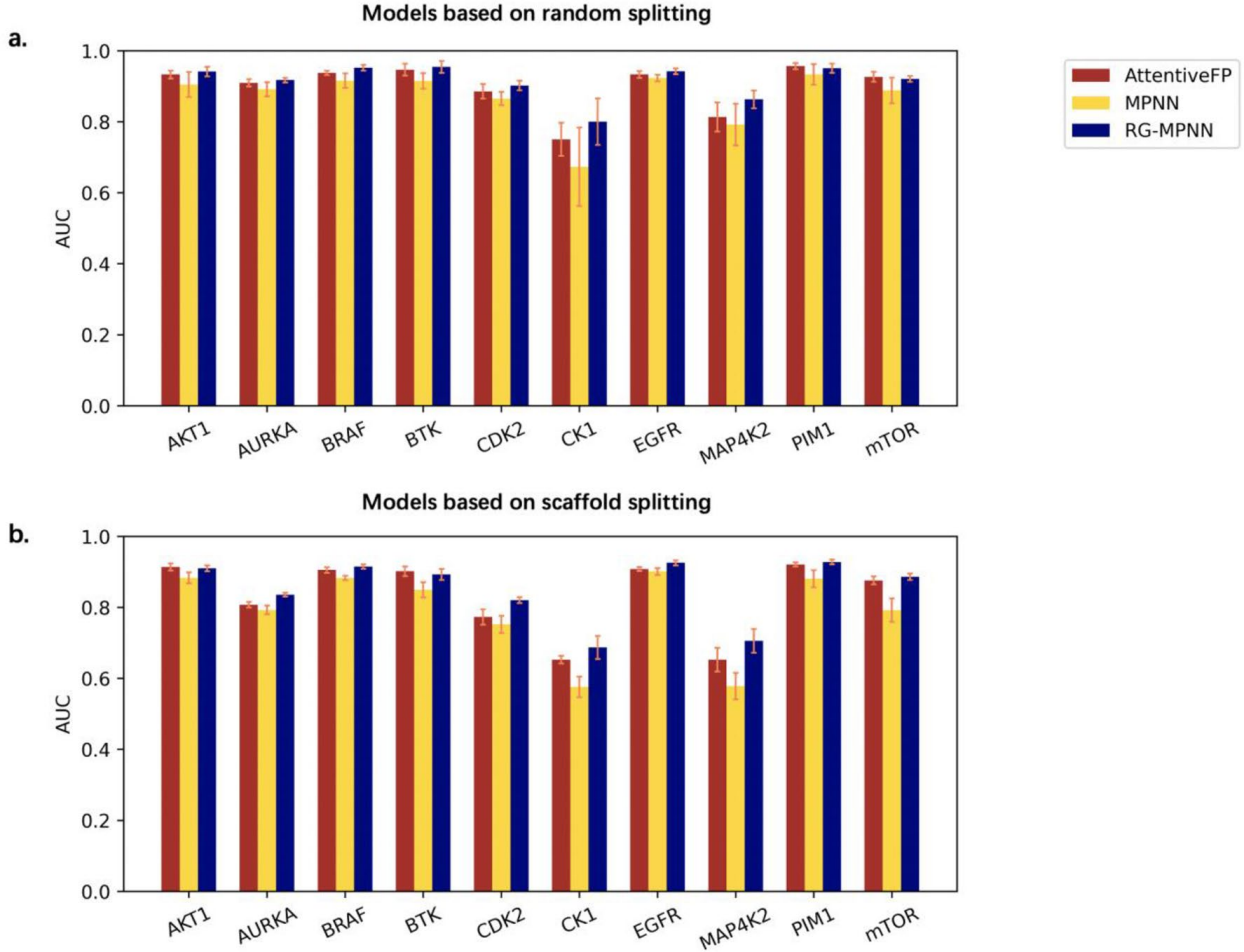
Model performance on kinase datasets in terms of AUC. **a** Model performance based on random splitting datasets. Our model RG-MPNN performs slightly better or comparable than AttentiveFP, better than MPNN model. **b** Model performance based on scaffold splitting datasets. Same trend is seen as (**a**), but performance is generally lower than that of (**a**)

It can be seen from Table 3 and Fig. 3 that the model tended to perform better on data sets with a relatively large number of molecules. On the other hand, it showed the limitation of GNNs for task prediction on small data sets, as the model did not perform well on the two small data sets of CK1 and MAP4K2. This limitation is mainly due to the relatively larger number of parameters of GNN that need to be trained and the parameters having not been fully trained would lead to underfitted models if the data set is too small. In addition, the data imbalance may be one another reason for the bad prediction performance. Nevertheless, RG-MPNN performed significantly better than AttentiveFP on these two tasks, and equally to or slightly better than AttentiveFP on most of other tasks.

On the models that based on scaffold split methods, we can see almost the same trend as that based on random split, but the scaffold split can be more challenging: the model performance is generally lower that the case of random split.

In addition, we also tested our models on other types of targets other than kinase targets, including four protein targets. These are datasets of published machine learning model results from our lab, the results show that our RG-MPNN model is comparable to traditional machine learning models, see Additional file 1: Table S8 for details, which is consistent to the evaluation of GNN and traditional machine learning on benchmark datasets.

### Applying reduced graph to MPNN architecture

From the tests on different data sets (benchmark data sets and kinase data sets), it has been proved that the RG-MPNN model is effective, which means that the MPNN architecture with RG pooling is effective when applied to a basic ResMPNN (see the previous methods section). In this part, we aim to explore the effect of applying this architecture to other models with MPNN architecture, to see whether the RG pooling can help MPNNs improve their predictive ability.

**Fig. 4:**
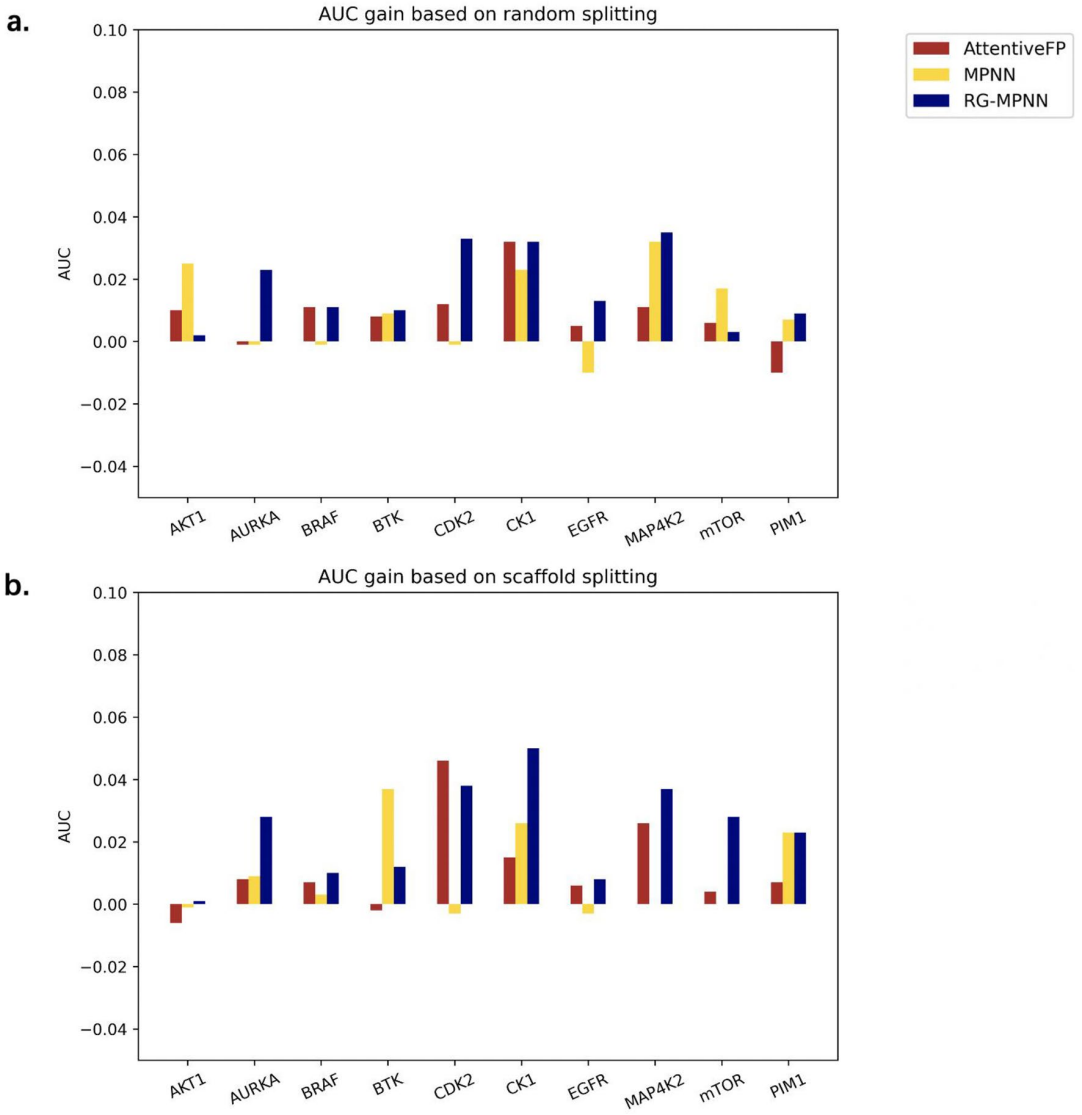
Effects on predictive performance that RG pooling brings to basis models. **a** AUC gains based on random splitting datasets. **b** AUC gains based on scaffold splitting datasets. Most of the AUC gains in both subfigures are positive, which means that the RG pooling is helpful to improve predictive performance of models

We compared three sets of models in pairs (ResMPNN *vs* RG-MPNN, AttentiveFP *vs* AttentiveFP with RG pooling, MPNN *vs* MPNN with RG pooling), each consisting of two models before and after being applied RG pooling. Totally, we built 600 models across ten kinase datasets, with two splitting methods (random and scaffold splitting) and repeated by five times. The detailed performance of each model is shown in Additional file 1: Table S9 and S10. The effects on prediction performance that RG pooling brings to basic models are shown in Fig. 4 (detailed difference can be seen in Additional file 1: Table S11 and S12), where the following points can be concluded: (a) the RG pooling can improve most basic models, including all the ResMPNNs, 80% of AttentiveFP models, and 55% of MPNNs, with the improvements of AUC ranging from − 0.010 to 0.046; (b) the RG pooling helps ResMPNN gain more improvement on scaffold splits than on random splits, while the gains of RG pooling for the other two models (AttentiveFP and MPNN), could not see a consistent trend between these two splitting methods. This indicated that the basic model ResMPNN is more compatible with the RG pooling.

Overall, from the experimental results, RG pooling can improve the models with MPNN architecture to varying degrees, increasing our prospects for applying this architecture in industry.

### Visualization and analysis of task-learned fingerprints

**Fig. 5:**
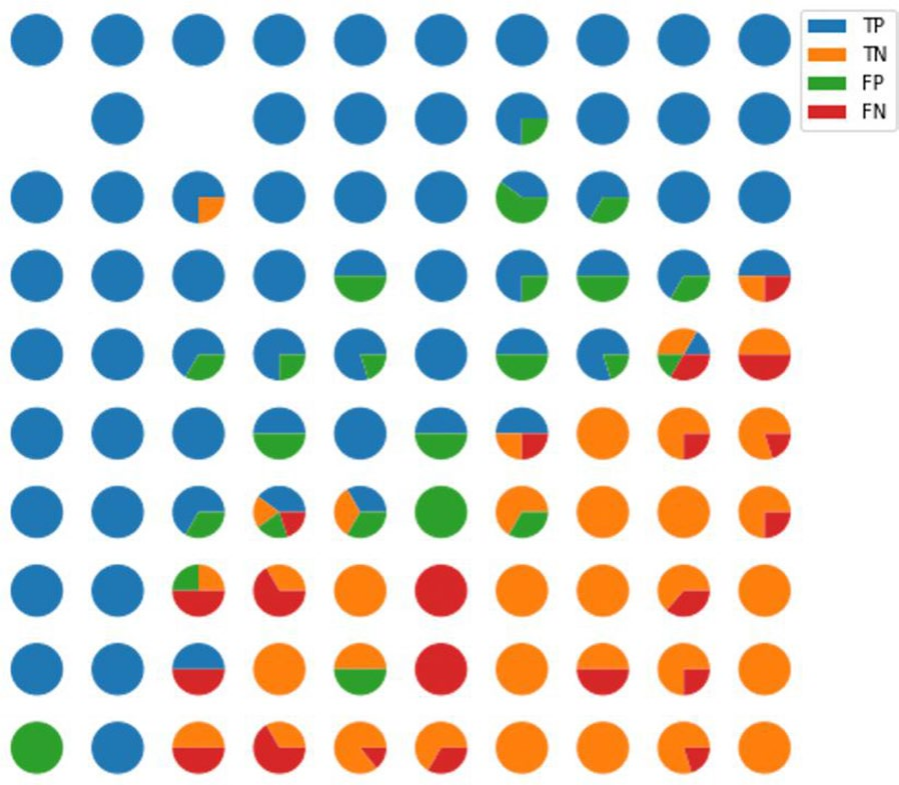
The SOM of the representation learned by RG-MPNN model on the AURKA bioactivity prediction task. Active (positive) molecules and inactive (negative) molecules are mapped to different zones, which means the representation learned by RG-MPNN has a good differentiating effect

We expect a good QSAR model not only to accurately predict the potential activity of each molecule, but also to help pharmacologists visually observe why some molecule is active (such as what substructure or property it has), and to measure the effect the structural differences on activity between two molecules. With this expectation, we extracted the hidden state of molecules in RG-MPNN as the task-learned representations and trained the representations into spatial arrangement via a self-organizing map (SOM) [50, 51] Fig. 5 shows the molecular representation distribution on a two-dimensional map where being projected to adjacent neurons means that two molecules are similar at the level of task-learned representation. It can be seen from the figure that active molecules and inactive molecules are mapped to different zones, with the diagonal line as the dividing line, the upper left corner mostly lies the active samples, and the lower right corner lies inactive ones. The conflicting neurons are concentrated near the diagonal, which is the junction of the two types of molecules. Molecules in this area are the most challenging ones to distinguish since these molecules have similar representations but different labels. Notably, prediction credibility of model is also implied in the map: the closer to the upper left and lower right corner, the higher the credibility to be active molecules and inactive ones, respectively.

Furthermore, to visually see the difference between similar molecules under different representations, we took two typical AURKA inhibitors (VX-680 and pha-739358) as examples and dive to look at their analogs under the task-learned representation and ECFP_4 [52] system, respectively (see Additional file 1: Figure S2 and S3). It can be concluded that in the two representation systems, the molecular structures in the same neuron are very similar, but different analogs are extracted. In terms of the consistency of molecular labels in the same neuron, the task-learned representation is better than ECFP, which is consistent with the previously observed phenomenon that the task-learned system has fewer conflicting neurons than the ECFP system. Strikingly, the task-learned system shows the possibility of completing scaffold hopping while ensuring activity.

### Activity interpretation for AURKA inhibitors

The task-learned representations are often criticized for being difficult to interpret, and it is difficult to gain intuitive knowledge from them, which is not conducive to the understanding of pharmacologists when applied to the practice of drug discovery. Therefore, we extracted the attention weights to learn the importance of each pharmacophore nodes, aiming at dig and provide some intuitive information to help drug development.

**Fig. 6:**
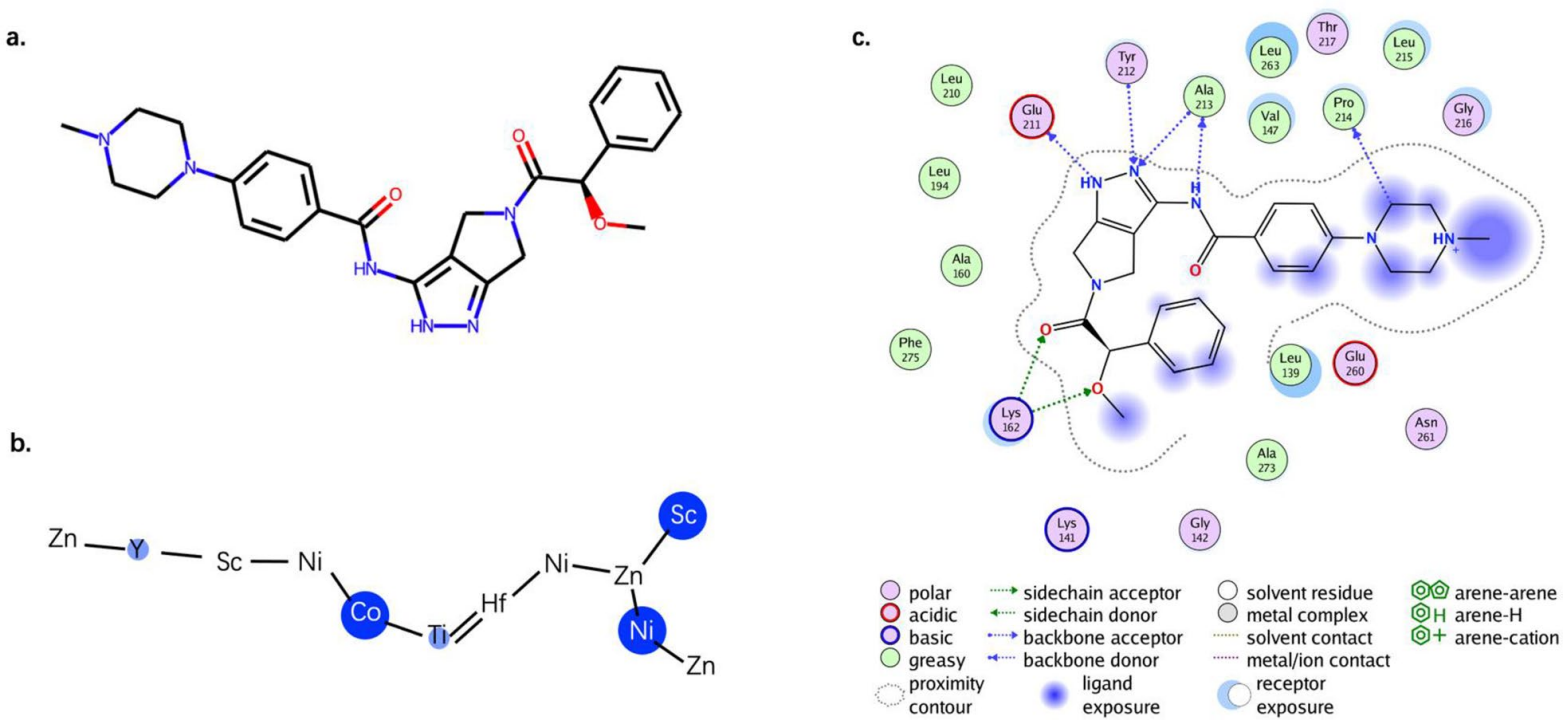
Explanation of the importance of pharmacophore or substructure. **a** The chemical structure of the pha-739358, an AURKA inhibitor. **b** The importance of pharmacophore learned by RG-MPNN. We can see that Y (aliphatic and positively ionizable), Co (acyclic and donor), Ti (aromatic and donor), Ni (acyclic and acceptor) and Sc (aromatic and no feature) play important roles in AURKA bioactivity. **c** Ligand-receptor interactions in the crystal complex (PDB ID: 2J50). Of note, Nitrogen atoms within Co and Ti form two important hydrogen bonds with receptor, which is consistent with the analysis of pharmacophore importance

Take the aforementioned pha-739358 (an AURKA inhibitor, shown in Fig. 6a as an example, we have annotated the degrees of the effect of the pharmacophore node in the molecule on the activity, as shown in Fig. 6b. We can see that Y (aliphatic and positively ionizable), Co (acyclic and donor), Ti (aromatic and donor), Ni (acyclic and acceptor) and Sc (aromatic and no feature) play important roles in molecular activity. Combining the ligand-receptor interaction diagram in the crystal complex (PDB ID: 2J50) for comparative analysis (Fig. 6c, we found that the two findings have a certain consistency. For example, Co and Ti, which form hydrogen bonds to the backbone, are labeled, which is consistent with the interactions appeared in the crystal complex. However, the findings are not completely overlapped, which is not surprising though, since one interpretation is derived from the interaction with receptor and the other from the knowledge from ligands. The interpretations of the two can be used for reference in practical applications. After all, in drug development practice, the more information from more perspectives, the more novel ideas can be provided, and the chance of discovering new drugs will increase.

## Conclusions

With the goal of integrating more prior chemical knowledge to establish predictive GNN models for chemical properties, we introduced a pharmacophore-based RG pooling method for MPNNs that can extract pharmacophore information hierarchically from molecular graphs. Therefore, in this work, we proposed the RG-MPNN model and compared it with the state-of-the-art GNN algorithms on the MoleculeNet benchmarks. The results showed that our models outperformed other models on ten out of twelve tasks. These results were also transferred to ten kinase data sets which were selected because they are representative kinase targets from each kinase family with great potential to be drug targets. Models built on these kinase data sets can be used in drug screening for inhibitors of these kinases. Furthermore, three groups of comparative experiments on the kinase data sets by applying the RG pooling were conducted, suggesting that this architecture can generally improve the predictive power of many MPNNs. It showed that this architecture had the potential to be extended to more MPNNs. We recommend readers to apply this architecture to their own MPNN model, not only because it is likely to improve its prediction accuracy, but also because the task-learned fingerprints obtained by the model bring the possibility of completing scaffold hopping while ensuring activity. Moreover, the fact that pharmacophore importance information can be quantified is in line with medical chemists’ intuitive needs and understanding needs in molecular design, which will help them gain insights for hit discovery and lead optimization.

## Supplementary Information

**Additional file 1:** **Table S1.** Basic information of the in-house datasets used in this work. **Table S2.** Atom features. **Table S3.** Bond features. **Figure S1.** Model performance on benchmark datasets in terms of AUC. **Table S4.** Molecular features and functional groups encoded by the SMARTS patterns used for pharmacophoric feature perception in RG generation. **Table S5.** General process of applying RG pooling for MPNNs. **Table S6.** Comparisons of the number of parameters and running time of the three models (RG-MPNN, AttentiveFP and MPNN). **Table S7.** Reported model performance on benchmark datasets. **Table S8.** Model performance on in-house datasets based on random splitting method. **Table S9.** Average model performance on kinase datasets based on random splitting method. **Table S10.** Average model performance on kinase datasets based on scaffold splitting method. **Table S11.** AUC gains based on random splitting datasets after adding RG-MPNN architecture. **Table S12.** AUC gains based on scaffold splitting datasets after adding RG-MPNN architecture. **Figure S2.** The SOM of the representation learned by RG-MPNN model on the AURKA bioactivity prediction task. It shows the molecules of the neurons where the VX-680 and the pha-739358 are located. **Figure S3.** The SOM of the ECFP_4 fingerprints for the AURKA inhibitors. It shows the molecules of the neurons where the VX-680 and the pha-739358 are located.

## Data Availability

The eleven benchmark data sets and ten kinase data sets are available at Github (https://github.com/ChloeKong/RG-MPNN). The code is available at GitHub (https://github.com/ChloeKong/RG-MPNN).

## Abbreviations

DNNs: Deep neural networks
QSAR/QSPR: Quantitative structure–activity (property) relationship
LBDD: Ligand-based drug design
ML: Machine learning
SVMs: Support vector machines
NB: Naïve Bayes
ANN: Artificial neural network
RF: Random forest
RNN: Recurrent neural network
GNN: Graph neural network
MPNN: Message-passing neural network
D-MPNN: Directed MPNN
R-GCN: Relational graph convolutional networks
GSN: Graph substructure networks
RGs: Reduced graphs
