# Supplementary material for: Integrating concept of pharmacophore with graph neural networks for chemical property prediction and interpretation
Source: J Cheminform. 2022 Aug 4;14:52. doi: 10.1186/s13321-022-00634-3 (PMC9351086; doi:10.1186/s13321-022-00634-3)
Supplement: Supplementary file 1 — Additional file 1: Table S1. Basic information of the in-house datasets used in this work. Table S2. Atom features. Table S3. Bond features. Figure S1. Model performance on benchmark datasets in terms of AUC. Table S4. Molecular features and functional groups encoded by the SMARTS patterns used for pharmacophoric feature perception in RG generation. Table S5. General process of applying RG pooling for MPNNs. Table S6. Comparisons of the number of parameters and running time of the three models (RG-MPNN, AttentiveFP and MPNN). Table S7. Reported model performance on benchmark datasets. Table S8. Model performance on in-house datasets based on random splitting method. Table S9. Average model performance on kinase datasets based on random splitting method. Table S10. Average model performance on kinase datasets based on scaffold splitting method. Table S11. AUC gains based on random splitting datasets after adding RG-MPNN architecture. Table S12. AUC gains based on scaffold splitting datasets after adding RG-MPNN architecture. Figure S2. The SOM of the representation learned by RG-MPNN model on the AURKA bioactivity prediction task. It shows the molecules of the neurons where the VX-680 and the pha-739358 are located. Figure S3. The SOM of the ECFP_4 fingerprints for the AURKA inhibitors. It shows the molecules of the neurons where the VX-680 and the pha-739358 are located. [file 13321_2022_634_MOESM1_ESM.pdf]

Supporting Information

Integrating concept of pharmacophore with Graph Neural Networks for chemical property prediction and interpretation

Yue Kong,[†,‡] Xiaoman Zhao,[†] Ruizi Liu,[†] Zhenwu Yang,[†] Hongyan Yin,[†,‡] Bowen Zhao,[‡] Jinling Wang,[‡] Bingjie Qin,[‡] Aixia Yan[*,†]

[†]State Key Laboratory of Chemical Resource Engineering, Department of Pharmaceutical Engineering, Beijing University of Chemical Technology, P. O. Box 53, 15 BeiSanHuan East Road, Beijing 100029, P. R. China.

[‡]Hyper-Dimension Insight Pharmaceuticals Ltd. Room 511, Block A, No. 2C, DongSanHuan North Road, Beijing, P. R. China.

[*] Corresponding author, Email: yanax@mail.buct.edu.cn

Table S1. Basic information of the in-house datasets used in this work

target name	short name	reference	total[a]	active	inactive	ratio[b]
hepatitis C virus (HCV) non-structural protein 3 (NS3) serine protease	HCV NS3	[S1]	413	287	126	0.69
GIIA secreted phospholipase A2 (GIIA sPLA2)	PLA2	[S2]	452	260	192	0.58
HIV-1 protease	HIV protease	[S3]	4855	2004	2851	0.41
Tyrosinase	Tyrosinase	[S4]	1097	375	722	0.34

a: number of molecules in total; b: active/total ratio.

Table S2. Atom features.

atom feature	size	description
atom type	100	type of atom (ex. C, N, O), by atomic number
# bonds	6	number of bonds the atom is involved in
formal charge	5	integer electronic charge assigned to atom
# Hs	5	number of bonded hydrogen atoms
hybridization	6	sp, sp2, sp3, sp3d, sp3d2 or other
aromaticity	1	whether this atom is part of an aromatic system
ring size	7	size of ring where the atom is involved in, 3-, 4-, 5-, 6-, 7- and 8-membered ring or ring size greater than 8

All features are one-hot encodings except for atomic mass, which is a real number scaled to be on the same order of magnitude.

Table S3. Bond features.

bond feature	size	description
bond type	4	single, double, triple, or aromatic
conjugated	1	whether the bond is conjugated
in ring	1	whether the bond is part of a ring
stereo	4	none, any, E/Z or cis/trans

All features are one-hot encodings.

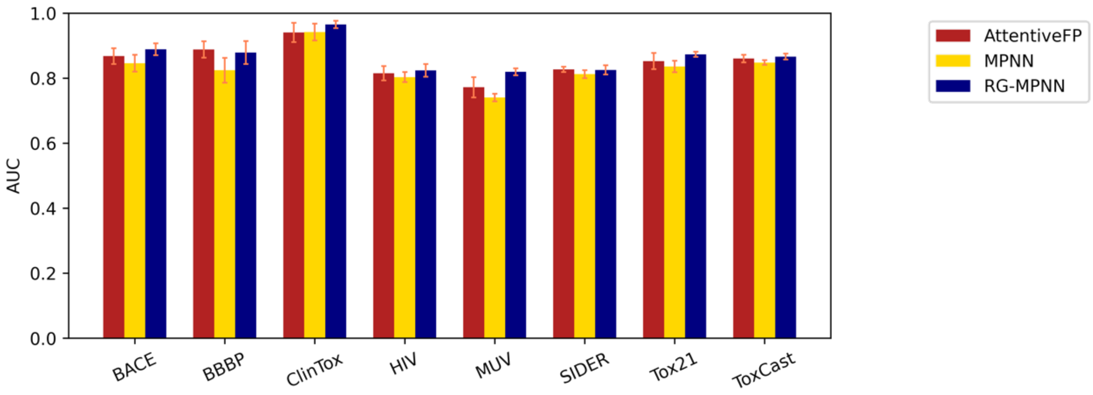

Figure S1. Model performance on benchmark datasets in terms of AUC.

Table S4. Molecular features and functional groups encoded by the SMARTS patterns used for pharmacophoric feature perception in RG generation.

Order of priority	Features	Rule descriptions
1	positively ionizable	Aliphatic basic amines
		amidine
		guanidine
		Imidazole
		Other functional groups which are likely to be protonated at physiological pH (7.4)
		Positive charge not directly adjacent to negative charge (exclusion of nitro-nitrogen)
2	Negatively ionizable	Carboxylic acid
		S- / P- acids (sulfonic, sulfonic, phosphonic or phosphinic acid)
		tetrazole
		acid imides
		Acid sulfonamides
		trifluoromethylsulfonamide
		Other functional groups which are likely to be deprotonated at physiological pH (7.4)
		Negative charge must not be directly adjacent to positive charge (exclusion of nitro-oxygen)
3	Donor/Acceptor	Both H-bond donor and acceptor features are present, refer to the definitions in the table for characteristics of the donor and acceptor
4	Acceptor	Every nitrogen, oxygen, and sulfur atom with at least one nondelocalized lonely electron pair without positive formal charge
		Exclusion of (sulfon) amide-N atoms due to delocalization

		Exclusion of amidine- / guanidine-N atoms due to positive charge and delocalization
5	Donor	Every nitrogen, oxygen, and sulfur atom having at least one covalently bound hydrogen atom without negative formal charge
6	No feature	Without any of the features described above

Adapted from reference [S5]

Table S5. General process of applying RG pooling for MPNNs

Atom-level Message-Passing
Given a molecule graph G, $atom \in G$, h_{atom}^{K} is the resulted hidden state of each atom after message passing for K time steps according to specific messaging rules or architecture in each MPNN model.
**Graph Reducing**
Each G corresponds to a unique RG, $RG = (V', E')$, V' represents the pharmacophore node, and E' represents the edge between pharmacophore nodes. Reduce graph according to predefined rules (Table S4) and the initial hidden state of pharmacophore node h_{rg}^{0} is achieved: $$h_{rg}^{0} = Reduce(\{h_{atom}^{K} \mid atom \in V'\})$$
**RG-level Message-Passing**
The message-passing at RG-level runs for T time steps, the message m_{rg}^{t+1} for each step and the hidden state of each pharmacophore node h_{rg}^{t+1} are achieved: $$m_{rg}^{t+1} = \sum_{rg' \in N(rg)} M_t\ (h_{rg}^{t}, h_{rg'}^{t})$$ $$h_{rg}^{t+1} = U_t\ (h_{rg}^{t}, m_{rg}^{t+1})$$
**Molecule Readout**
Finally, the whole molecule embedding h_{mol} and the final prediction of molecular property are achieved: $$h_{mol} = R(\{h_{rg}^{T} \mid V' \in RG\})$$ $$\hat{y} = MLP(h_{mol})$$

Table S6. Comparisons of the number of parameters and running time of the three models (RG-MPNN, AttentiveFP and MPNN)

Model name	Number of parameters	Average running time for 10 epochs (tested on AURKA dataset)
AttentiveFP	1,882,113	36.85s
MPNN	9,867,137	60.04s
RG-MPNN	1,942,785	37.35s

Table S7. Reported model performance on benchmark datasets.

Category	Dataset	# Compounds	Task Type	# Tasks	Metrics	AttentiveFP[a]	MPNN[b]	GC[b]	Weave[b]	D-MPNN[c]	SVM[d]	XGBoost[d]	RF[d]
Physical chemistry	ESOL	1128	Regression	1	RMSE	0.503 ± 0.076	0.580 ± 0.030	0.970 ± 0.010	0.610 ± 0.070	0.665 ± 0.052	0.516	0.571	0.631
	FreeSolv	642	Regression	1	RMSE	0.736 ± 0.037	1.150 ± 0.120	1.400 ± 0.160	1.220 ± 0.280	1.167 ± 0.150	0.674	0.707	0.888
	Lipophilicity	4200	Regression	1	RMSE	0.578 ± 0.018	0.719 ± 0.031	0.655 ± 0.036	0.715 ± 0.035	0.596 ± 0.050	0.567	0.556	0.649
bioactivity	MUV	93087	Classification	17	ROC-AUC	0.843±0.012	-	-	-	-	-	-	-
	HIV	41127	Classification	1	ROC-AUC	0.832 ± 0.021	-	0.763 ± 0.016	0.703 ± 0.039	0.816 ± 0.023	0.840	0.848	0.846
	BACE	1513	Classification	1	ROC-AUC	0.850 ± 0.012	-	0.783 ± 0.014	0.806 ± 0.002	0.878 ± 0.032	0.861	0.889	0.861
Physiology or toxicity	BBBP	2039	Classification	1	ROC-AUC	0.920 ± 0.015	-	0.690 ± 0.009	0.671 ± 0.014	0.913 ± 0.026	0.899	0.886	0.907
	Tox21	7831	Classification	12	ROC-AUC	0.858 ± 0.014	-	0.829 ± 0.006	0.820 ± 0.010	0.845 ± 0.015	0.826	0.847	0.858
	ToxCast	8575	Classification	617	ROC-AUC	0.805±0.022	-	0.716 ± 0.014	0.742 ± 0.003	0.737 ± 0.013	0.724	0.773	0.782
	SIDER	1427	Classification	27	ROC-AUC	0.637 ± 0.017	-	0.638 ± 0.012	0.581 ± 0.027	0.646 ± 0.016	0.620	0.665	0.659
	ClinTox	1478	Classification	2	ROC-AUC	0.940 ± 0.018	-	0.807 ± 0.047	0.832 ± 0.037	0.894 ± 0.027	0.966	0.919	0.964

a: derived from reference [S6]; b: derived from reference [S7]; c: derived from reference [S8]; d: derived from reference [S9].

Table S8. Model performance on in-house datasets based on random splitting method

dataset	model	Test_Acc	Test_MCC
HCV NS3	MPNN	0.771	0.409
	AttentiveFP	0.8	0.511
	RG-MPNN	0.871	0.698
	**Reported best model (SVM + ECFP_4) [S1]**	**0.907**	**-**
PLA2	MPNN	0.811	0.624
	AttentiveFP	0.889	0.777
	**RG-MPNN**	**0.912**	**0.832**
	Reported best model (SVM + ECFP_4) [S2]	0.907	0.820
HIV protease	MPNN	0.783	0.568
	AttentiveFP	0.824	0.643
	**RG-MPNN**	**0.835**	**0.660**
	Reported best model (SVM + MACCS) [S3]	0.831	-
Tyrosinase	MPNN	0.817	0.597
	AttentiveFP	0.821	0.599
	RG-MPNN	0.849	0.669
	**Reported best model (MLP + ECFP_4) [S4]**	**0.914**	**0.81**

Note best model performance is in bold.

Table S9. Average model performance on kinase datasets based on random splitting method

dataset	model	te_AUC	te_MCC	te_ACC	te_SE	te_SP	tra_AUC	tra_MCC	tra_ACC	tra_SE	tra_SP	val_AUC	val_MCC	val_ACC	val_SE	val_SP
EGFR	MPNN	0.9232	0.6988	0.8494	0.8614	0.8368	0.972	0.8226	0.9116	0.9132	0.9096	0.934	0.7176	0.8578	0.8634	0.8534
	MPNN with RG pooling	0.9136	0.6824	0.841	0.8478	0.8338	0.9518	0.775	0.8874	0.9	0.874	0.9222	0.7008	0.85	0.8488	0.8522
	AttentiveFP	0.933	0.729	0.8642	0.8852	0.842	0.9862	0.892	0.9458	0.949	0.9426	0.9396	0.7402	0.8702	0.8792	0.8606
	AttentiveFP with RG pooling	0.9376	0.7404	0.8698	0.871	0.8692	0.976	0.8342	0.9172	0.919	0.9156	0.9408	0.7302	0.8646	0.8572	0.8734
	ResMPNN	0.929	0.7156	0.8572	0.875	0.839	0.9844	0.8832	0.9416	0.9428	0.9404	0.9406	0.7456	0.8732	0.8764	0.8692
	RG-MPNN	0.942	0.7378	0.8686	0.874	0.8632	0.9678	0.804	0.9024	0.9032	0.9006	0.9408	0.7432	0.8714	0.862	0.8812
BRAF	MPNN	0.9158	0.7028	0.894	0.9364	0.7568	0.9776	0.8548	0.9474	0.9738	0.8654	0.9302	0.7118	0.8972	0.9374	0.765
	MPNN with RG pooling	0.9152	0.686	0.8904	0.9446	0.7152	0.9654	0.8136	0.9332	0.9684	0.8224	0.9352	0.7238	0.9036	0.9554	0.7356
	AttentiveFP	0.9378	0.7532	0.9124	0.9502	0.7894	0.9928	0.9166	0.9698	0.9824	0.9296	0.9668	0.7912	0.9256	0.9612	0.8118
	AttentiveFP with RG pooling	0.9492	0.748	0.9108	0.9506	0.7804	0.9936	0.916	0.9692	0.9812	0.9314	0.962	0.7924	0.9258	0.959	0.8198
	ResMPNN	0.9406	0.7536	0.9116	0.9432	0.8082	0.9932	0.9182	0.97	0.9824	0.9316	0.9664	0.7932	0.9256	0.9558	0.8306
	RG-MPNN	0.952	0.7742	0.9194	0.9504	0.8192	0.9838	0.8554	0.9476	0.9706	0.8758	0.965	0.805	0.9308	0.9662	0.8164
PIM1	MPNN	0.9332	0.7072	0.8856	0.9196	0.7918	0.9854	0.8646	0.9466	0.9596	0.9104	0.9596	0.759	0.9084	0.933	0.8328
	MPNN with RG pooling	0.9406	0.719	0.8884	0.9154	0.8138	0.9724	0.811	0.9246	0.9386	0.8858	0.9602	0.7532	0.9046	0.9302	0.8306
	AttentiveFP	0.9568	0.732	0.8966	0.9334	0.792	0.9898	0.8876	0.9554	0.9648	0.9294	0.9676	0.7806	0.9178	0.9516	0.818
	AttentiveFP with RG pooling	0.9472	0.7292	0.8932	0.9204	0.8176	0.9896	0.8958	0.9584	0.9652	0.9392	0.9726	0.809	0.9274	0.9472	0.8668
	ResMPNN	0.9412	0.7182	0.8902	0.9258	0.793	0.9862	0.8726	0.95	0.9636	0.9124	0.9584	0.777	0.9166	0.9462	0.8274
	RG-MPNN	0.9506	0.7506	0.9028	0.9342	0.816	0.9846	0.8588	0.944	0.9576	0.9084	0.9668	0.7754	0.9164	0.9492	0.818
mTOR	MPNN	0.8884	0.5908	0.851	0.8658	0.7874	0.9378	0.6716	0.8836	0.8922	0.847	0.9246	0.6238	0.8566	0.8588	0.852
	MPNN with RG pooling	0.9052	0.601	0.8468	0.8536	0.8196	0.9302	0.649	0.8704	0.8742	0.8548	0.9334	0.652	0.869	0.8706	0.8658
	AttentiveFP	0.9266	0.662	0.8878	0.9156	0.7714	0.9852	0.8354	0.9442	0.945	0.9402	0.9604	0.7214	0.9072	0.9264	0.8332
	AttentiveFP with RG pooling	0.933	0.6488	0.8792	0.9008	0.789	0.9758	0.7794	0.9234	0.9246	0.9186	0.9564	0.7266	0.9102	0.9308	0.8282
	ResMPNN	0.918	0.6408	0.8838	0.9194	0.7376	0.9772	0.815	0.937	0.9392	0.9268	0.9532	0.708	0.9066	0.933	0.7936

	RG-MPNN	0.9206	0.6296	0.8546	0.8538	0.8516	0.964	0.7358	0.9068	0.9096	0.8958	0.9534	0.6802	0.8798	0.882	0.8764
AKT1	MPNN	0.905	0.669	0.8342	0.8626	0.8026	0.9394	0.7614	0.882	0.9198	0.8352	0.9144	0.7076	0.853	0.902	0.7988
	MPNN with RG pooling	0.9304	0.7364	0.8688	0.8924	0.8412	0.9662	0.8136	0.9076	0.938	0.8702	0.9424	0.7518	0.8758	0.904	0.8456
	AttentiveFP	0.933	0.751	0.876	0.916	0.829	0.9854	0.882	0.9418	0.9588	0.921	0.9536	0.788	0.894	0.929	0.8538
	AttentiveFP with RG pooling	0.9426	0.7772	0.8882	0.9284	0.8416	0.9882	0.9084	0.9548	0.9656	0.9414	0.958	0.7928	0.8954	0.9456	0.8396
	ResMPNN	0.9396	0.7564	0.8792	0.9014	0.852	0.9732	0.8344	0.9178	0.943	0.8872	0.9528	0.7716	0.8856	0.9232	0.8438
	RG-MPNN	0.9414	0.7706	0.8862	0.9092	0.8584	0.9756	0.8464	0.9238	0.9458	0.897	0.9534	0.7758	0.888	0.9176	0.8548
AURKA	MPNN	0.8916	0.634	0.8238	0.8934	0.7248	0.9564	0.7884	0.8982	0.9278	0.8546	0.8952	0.6266	0.8204	0.8908	0.7178
	MPNN with RG pooling	0.891	0.6294	0.8212	0.8772	0.7424	0.9386	0.729	0.8698	0.9092	0.812	0.892	0.6282	0.8214	0.8836	0.733
	AttentiveFP	0.9094	0.6646	0.8384	0.8852	0.77	0.9858	0.8896	0.9468	0.9628	0.9236	0.9144	0.6642	0.839	0.8768	0.7816
	AttentiveFP with RG pooling	0.9086	0.6714	0.8422	0.8876	0.776	0.9658	0.8322	0.9192	0.9446	0.8826	0.9176	0.6636	0.8376	0.8732	0.7844
	ResMPNN	0.8946	0.6488	0.831	0.8736	0.7676	0.9666	0.832	0.9192	0.9448	0.8822	0.9102	0.6794	0.8448	0.876	0.7978
	RG-MPNN	0.9172	0.6902	0.8506	0.8924	0.7896	0.9802	0.8654	0.9352	0.955	0.9066	0.9178	0.6782	0.8452	0.8716	0.8034
BTK	MPNN	0.9148	0.67	0.867	0.9264	0.7266	0.9638	0.8138	0.9254	0.9636	0.8294	0.9298	0.6942	0.8766	0.9328	0.741
	MPNN with RG pooling	0.924	0.7018	0.8814	0.9404	0.7336	0.9592	0.7856	0.9138	0.9544	0.8126	0.9504	0.736	0.892	0.9416	0.773
	AttentiveFP	0.9468	0.7476	0.8994	0.9556	0.7626	0.9856	0.8822	0.9524	0.9772	0.8912	0.96	0.7674	0.9056	0.9522	0.7918
	AttentiveFP with RG pooling	0.9548	0.7674	0.9062	0.9476	0.806	0.9858	0.8796	0.951	0.9762	0.8892	0.9634	0.7904	0.914	0.9478	0.8304
	ResMPNN	0.9444	0.724	0.8876	0.9264	0.7952	0.9598	0.7986	0.919	0.9564	0.8252	0.9486	0.7476	0.896	0.9372	0.7966
	RG-MPNN	0.9544	0.7586	0.9038	0.952	0.784	0.9842	0.8694	0.9474	0.9724	0.8842	0.962	0.804	0.9206	0.9622	0.818
CDK2	MPNN	0.8652	0.5672	0.7828	0.8178	0.747	0.9582	0.7802	0.89	0.8888	0.8912	0.8914	0.6048	0.8016	0.8138	0.79
	MPNN with RG pooling	0.8638	0.5782	0.7888	0.7982	0.778	0.962	0.7964	0.8982	0.9074	0.8884	0.8732	0.6142	0.8072	0.81	0.803
	AttentiveFP	0.8856	0.6236	0.8118	0.8144	0.8088	0.9628	0.8096	0.9048	0.905	0.9042	0.8912	0.614	0.8062	0.8076	0.8054
	AttentiveFP with RG pooling	0.8972	0.6518	0.8252	0.8182	0.833	0.9868	0.8802	0.94	0.939	0.941	0.9	0.6578	0.8284	0.8124	0.845
	ResMPNN	0.869	0.5892	0.7948	0.7904	0.798	0.9794	0.8662	0.933	0.94	0.9256	0.886	0.6138	0.8062	0.836	0.777
	RG-MPNN	0.902	0.6524	0.8252	0.8552	0.7942	0.973	0.8236	0.9118	0.9174	0.9064	0.902	0.646	0.8214	0.857	0.7876

MAP4K2	MPNN	0.792	0.4566	0.7618	0.6122	0.8336	0.89	0.6318	0.8422	0.757	0.8812	0.8444	0.437	0.746	0.6114	0.8184
	MPNN with RG pooling	0.8236	0.516	0.7866	0.693	0.8286	0.9586	0.777	0.9	0.8912	0.904	0.8286	0.5038	0.7728	0.7352	0.7894
	AttentiveFP	0.8132	0.484	0.771	0.6462	0.8324	0.9176	0.7024	0.875	0.7858	0.9154	0.8296	0.4974	0.7822	0.6802	0.822
	AttentiveFP with RG pooling	0.824	0.454	0.746	0.6922	0.775	0.9548	0.767	0.8952	0.8752	0.905	0.8344	0.4748	0.7618	0.747	0.7678
	ResMPNN	0.8276	0.504	0.7686	0.726	0.788	0.9544	0.744	0.8874	0.8548	0.902	0.8588	0.562	0.8	0.7584	0.8236
	RG-MPNN	0.863	0.5398	0.791	0.719	0.826	0.9646	0.7826	0.906	0.8662	0.924	0.8468	0.5082	0.7754	0.7102	0.803
CK1	MPNN	0.6728	0.1564	0.79	0.1922	0.943	0.741	0.2448	0.8212	0.2868	0.9464	0.8032	0.1856	0.7928	0.2534	0.9262
	MPNN with RG pooling	0.6958	0	0.795	0	1	0.608	0	0.8098	0	1	0.8054	0	0.8048	0	1
	AttentiveFP	0.7506	0.3132	0.7954	0.3884	0.8992	0.9016	0.5984	0.8878	0.5884	0.9588	0.8476	0.4022	0.8224	0.45	0.913
	AttentiveFP with RG pooling	0.7822	0.2872	0.815	0.2122	0.9686	0.83	0.4216	0.853	0.3556	0.9708	0.8492	0.3068	0.8226	0.2328	0.9658
	ResMPNN	0.768	0.2892	0.8076	0.3366	0.923	0.885	0.575	0.8868	0.5818	0.959	0.8995	0.49775	0.847	0.56775	0.915
	RG-MPNN	0.8002	0.4326	0.8328	0.4338	0.937	0.878	0.553	0.875	0.5386	0.9532	0.8412	0.424	0.8374	0.424	0.938

Table S10. Average model performance on kinase datasets based on scaffold splitting method

dataset	model	te_AUC	te_MCC	te_ACC	te_SE	te_SP	tra_AUC	tra_MCC	tra_ACC	tra_SE	tra_SP	val_AUC	val_MCC	val_ACC	val_SE	val_SP
EGFR	MPNN	0.901	0.648	0.8252	0.8336	0.8194	0.9454	0.7426	0.8722	0.8814	0.861	0.8996	0.6256	0.8126	0.8362	0.7946
	MPNN with RG pooling	0.8978	0.639	0.8226	0.8006	0.8386	0.9158	0.6802	0.8408	0.865	0.813	0.9002	0.6424	0.8228	0.8224	0.8228
	AttentiveFP	0.908	0.6842	0.8452	0.8212	0.8626	0.98	0.8624	0.9316	0.9346	0.9282	0.9118	0.6686	0.8366	0.817	0.852
	AttentiveFP with RG pooling	0.9144	0.6734	0.836	0.8658	0.8142	0.9668	0.802	0.9016	0.9096	0.8926	0.9222	0.6708	0.8352	0.8566	0.8194
	ResMPNN	0.917	0.6688	0.8362	0.8366	0.8354	0.9584	0.783	0.8922	0.898	0.8852	0.9098	0.6522	0.8272	0.8326	0.823
	RG-MPNN	0.9254	0.706	0.8542	0.8646	0.8462	0.957	0.7748	0.888	0.895	0.88	0.9226	0.6816	0.8408	0.8626	0.8242
BRAF	MPNN	0.883	0.614	0.8368	0.9116	0.6764	0.9586	0.7732	0.9234	0.9582	0.8004	0.8932	0.6254	0.8362	0.909	0.6916
	MPNN with RG pooling	0.8856	0.6106	0.8364	0.9216	0.6538	0.9512	0.7772	0.9262	0.9668	0.7826	0.89	0.625	0.8368	0.9246	0.6636
	AttentiveFP	0.9054	0.6852	0.8662	0.926	0.7382	0.987	0.8798	0.9592	0.9766	0.8964	0.9156	0.6808	0.8604	0.9278	0.7274
	AttentiveFP with RG pooling	0.9124	0.6806	0.8632	0.9152	0.7518	0.9922	0.897	0.965	0.9802	0.9102	0.905	0.6538	0.849	0.9156	0.717
	ResMPNN	0.9046	0.6762	0.8624	0.9226	0.733	0.9896	0.8936	0.964	0.9802	0.906	0.9162	0.6878	0.8634	0.927	0.7376
	RG-MPNN	0.915	0.6768	0.8622	0.919	0.7396	0.9832	0.857	0.9518	0.9732	0.8738	0.9144	0.6668	0.854	0.9174	0.7286
PIM1	MPNN	0.8808	0.6116	0.8178	0.8668	0.7384	0.9674	0.7796	0.9204	0.9462	0.8354	0.913	0.6714	0.8458	0.9054	0.7514
	MPNN with RG pooling	0.9036	0.6574	0.8368	0.8574	0.8036	0.9734	0.8176	0.934	0.9544	0.8682	0.9138	0.6822	0.85	0.889	0.788
	AttentiveFP	0.9208	0.6814	0.8458	0.8486	0.8414	0.9878	0.869	0.9526	0.9672	0.9052	0.9374	0.7314	0.8724	0.906	0.8202
	AttentiveFP with RG pooling	0.9282	0.7048	0.8588	0.8736	0.8356	0.9896	0.884	0.958	0.97	0.9192	0.929	0.715	0.866	0.9114	0.794
	ResMPNN	0.9046	0.6658	0.8434	0.8978	0.756	0.9714	0.802	0.9286	0.952	0.8528	0.9256	0.6932	0.856	0.9336	0.7326
	RG-MPNN	0.9278	0.7096	0.8614	0.8758	0.8378	0.9882	0.8722	0.9536	0.9662	0.9126	0.9344	0.7216	0.8684	0.911	0.801
mTOR	MPNN	0.7922	0.4082	0.738	0.7676	0.6636	0.9324	0.6432	0.873	0.8774	0.8528	0.843	0.506	0.7788	0.7826	0.7684
	MPNN with RG pooling	0.7922	0.3942	0.714	0.7172	0.7056	0.9272	0.6168	0.8664	0.8736	0.8322	0.8418	0.475	0.7564	0.753	0.7664
	AttentiveFP	0.8758	0.5878	0.8272	0.863	0.7364	0.976	0.7742	0.927	0.9292	0.9158	0.908	0.641	0.8506	0.8646	0.812
	AttentiveFP with RG pooling	0.8798	0.6076	0.8378	0.877	0.7382	0.9882	0.8426	0.9516	0.954	0.9402	0.9106	0.63	0.8532	0.8884	0.7544

	ResMPNN	0.8584	0.5142	0.7984	0.8468	0.6766	0.9596	0.7152	0.9074	0.9136	0.8764	0.9058	0.6084	0.8398	0.8652	0.7682
	RG-MPNN	0.8862	0.5742	0.8168	0.8402	0.7584	0.9646	0.7304	0.9122	0.9168	0.888	0.915	0.6478	0.8522	0.8624	0.8238
AKT1	MPNN	0.883	0.6052	0.8042	0.8346	0.7858	0.9454	0.7752	0.891	0.946	0.8132	0.8776	0.6092	0.803	0.8468	0.774
	MPNN with RG pooling	0.8822	0.5872	0.8032	0.768	0.824	0.9628	0.8078	0.9072	0.9432	0.8556	0.8996	0.6566	0.8334	0.8142	0.8462
	AttentiveFP	0.9138	0.6694	0.8396	0.8472	0.8348	0.9808	0.86	0.9324	0.9564	0.898	0.924	0.7026	0.8536	0.8662	0.8452
	AttentiveFP with RG pooling	0.9078	0.6678	0.841	0.825	0.8504	0.988	0.8964	0.9498	0.9676	0.9244	0.9248	0.7258	0.8666	0.857	0.8732
	ResMPNN	0.9088	0.6472	0.8224	0.8722	0.7926	0.9694	0.822	0.9138	0.9504	0.862	0.9286	0.6956	0.8424	0.9144	0.7938
	RG-MPNN	0.91	0.6364	0.8204	0.8512	0.8016	0.97	0.819	0.9124	0.9452	0.8658	0.918	0.69	0.8442	0.882	0.819
AURKA	MPNN	0.7928	0.471	0.7336	0.7678	0.7034	0.9618	0.8016	0.9066	0.9452	0.8454	0.8416	0.5582	0.781	0.8182	0.7362
	MPNN with RG pooling	0.8016	0.4702	0.7326	0.76	0.7084	0.9606	0.8034	0.9072	0.947	0.8446	0.8586	0.5626	0.7822	0.8108	0.7492
	AttentiveFP	0.8072	0.4746	0.7368	0.7444	0.7302	0.9442	0.74	0.8776	0.9148	0.8186	0.8568	0.5544	0.777	0.7778	0.7762
	AttentiveFP with RG pooling	0.8148	0.507	0.7536	0.7432	0.7626	0.9696	0.812	0.9114	0.9452	0.8576	0.8622	0.5784	0.79	0.7942	0.7852
	ResMPNN	0.8074	0.4908	0.7422	0.7934	0.6964	0.964	0.8088	0.9098	0.947	0.851	0.8496	0.5546	0.778	0.801	0.751
	RG-MPNN	0.8358	0.5222	0.759	0.7908	0.7308	0.9814	0.8764	0.9418	0.964	0.9062	0.874	0.6212	0.812	0.8398	0.7794
BTK	MPNN	0.8492	0.5452	0.7866	0.8434	0.695	0.938	0.7358	0.8986	0.9444	0.7716	0.8812	0.6344	0.8282	0.884	0.7394
	MPNN with RG pooling	0.8864	0.5982	0.8126	0.8878	0.6908	0.9608	0.7908	0.9206	0.9636	0.8004	0.9334	0.6948	0.8546	0.9032	0.7778
	AttentiveFP	0.9016	0.6696	0.845	0.8958	0.7628	0.9746	0.8336	0.9364	0.9674	0.85	0.9422	0.7538	0.883	0.9046	0.8486
	AttentiveFP with RG pooling	0.8998	0.643	0.8324	0.8778	0.7588	0.9702	0.8242	0.9328	0.9684	0.8342	0.9416	0.721	0.8656	0.884	0.8362
	ResMPNN	0.8802	0.583	0.8062	0.8878	0.674	0.98	0.8536	0.944	0.976	0.8558	0.9402	0.7274	0.8718	0.9276	0.7838
	RG-MPNN	0.8926	0.6392	0.8314	0.8852	0.7442	0.9806	0.8498	0.9426	0.9726	0.8588	0.9432	0.7536	0.8828	0.9032	0.8504
CDK2	MPNN	0.752	0.3764	0.7052	0.6234	0.7534	0.949	0.7582	0.88	0.8984	0.8584	0.8252	0.4676	0.7372	0.7622	0.7244
	MPNN with RG pooling	0.7488	0.3708	0.6932	0.6746	0.7046	0.944	0.7414	0.8718	0.892	0.8478	0.8024	0.4388	0.7212	0.7598	0.7016
	AttentiveFP	0.773	0.412	0.72	0.664	0.7534	0.966	0.8182	0.9098	0.9206	0.8968	0.8252	0.4514	0.7234	0.7812	0.6946
	AttentiveFP with RG pooling	0.8186	0.4918	0.7614	0.6852	0.8062	0.9658	0.815	0.908	0.9174	0.8974	0.8284	0.4682	0.7402	0.7622	0.729
	ResMPNN	0.7828	0.4094	0.7202	0.6466	0.7634	0.9612	0.7994	0.9002	0.9162	0.8816	0.8108	0.4442	0.7234	0.76	0.7054

	RG-MPNN	0.8204	0.4948	0.7598	0.7106	0.7886	0.9664	0.802	0.9018	0.9184	0.8824	0.8278	0.5016	0.7568	0.7814	0.7444
MAP4K2	MPNN	0.5782	0.1742	0.6876	0.2846	0.854	0.8328	0.4912	0.7954	0.6334	0.8676	0.7508	0.219	0.6576	0.3438	0.84
	MPNN with RG pooling	0.578	0.1346	0.6448	0.3846	0.7526	0.7962	0.4662	0.7766	0.6162	0.8484	0.7348	0.2496	0.6668	0.425	0.8074
	AttentiveFP	0.6524	0.2774	0.6854	0.5384	0.7462	0.9558	0.7456	0.888	0.859	0.9012	0.7865	0.34325	0.68975	0.6015	0.741
	AttentiveFP with RG pooling	0.6786	0.2926	0.7258	0.408	0.8572	0.966	0.8166	0.9174	0.9158	0.9182	0.7824	0.4214	0.7402	0.5248	0.8654
	ResMPNN	0.669	0.2808	0.7284	0.3616	0.8794	0.9898	0.887	0.9504	0.9492	0.9508	0.811	0.4972	0.7724	0.575	0.8872
	RG-MPNN	0.7056	0.3058	0.7258	0.4388	0.8446	0.967	0.8376	0.9284	0.9122	0.9356	0.7846	0.454	0.752	0.5624	0.8618
CK1	MPNN	0.5756	-0.0198	0.7114	0.1572	0.8348	0.8416	0.4808	0.8448	0.5304	0.9216	0.759	0.1938	0.7576	0.3572	0.8424
	MPNN with RG pooling	0.6016	0.018	0.7322	0.1716	0.8572	0.7904	0.3954	0.8362	0.373	0.9492	0.708	0.1458	0.7446	0.3002	0.8392
	AttentiveFP	0.6526	0.1592	0.7712	0.2572	0.8858	0.9638	0.7704	0.9294	0.792	0.963	0.8396	0.3522	0.7974	0.5142	0.8578
	AttentiveFP with RG pooling	0.6674	0.3384	0.8232	0.3288	0.9332	0.94025	0.721	0.91575	0.726	0.962	0.8158	0.3276	0.8026	0.4428	0.8786
	ResMPNN	0.6368	0.1768	0.7946	0.2286	0.9208	0.9968	0.9336	0.979	0.954	0.9848	0.8678	0.449	0.8474	0.5002	0.9212
	RG-MPNN	0.687	0.3332	0.8128	0.4002	0.9048	0.9716	0.792	0.9358	0.8126	0.9658	0.8398	0.42	0.8122	0.6	0.8576

Table S11. AUC gains based on random splitting datasets after adding RG-MPNN architecture.

pair comparison	AKT1	AURKA	BRAF	BTK	CDK2	CK1	EGFR	MAP4K2	mTOR	PIM1
MPNN with RG pooling vs MPNN	0.025	-0.001	-0.001	0.009	-0.001	0.023	-0.010	0.032	0.017	0.007
AttentiveFP with RG pooling vs AttentiveFP	0.010	-0.001	0.011	0.008	0.012	0.032	0.005	0.011	0.006	-0.010
RG-MPNN vs ResMPNN	0.002	0.023	0.011	0.010	0.033	0.032	0.013	0.035	0.003	0.009

Table S12. AUC gains based on scaffold splitting datasets after adding RG-MPNN architecture.

pair comparison	AKT1	AURKA	BRAF	BTK	CDK2	CK1	EGFR	MAP4K2	mTOR	PIM1
MPNN with RG pooling vs MPNN	-0.001	0.009	0.003	0.037	-0.003	0.026	-0.003	0	0	0.023
AttentiveFP with RG pooling vs AttentiveFP	-0.006	0.008	0.007	-0.002	0.046	0.015	0.006	0.026	0.004	0.007
RG-MPNN vs ResMPNN	0.001	0.028	0.010	0.012	0.038	0.050	0.008	0.037	0.028	0.023

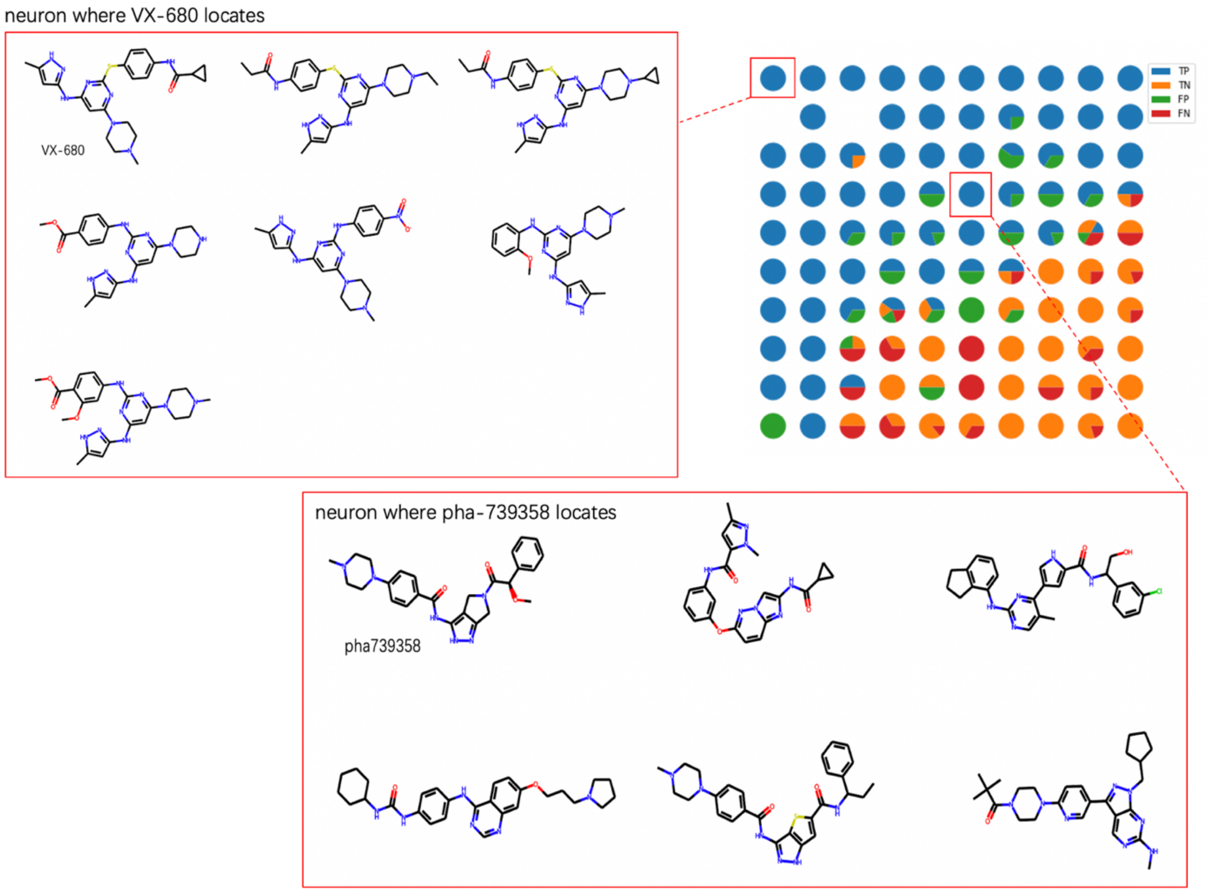

Figure S2. The SOM of the representation learned by RG-MPNN model on the AURKA bioactivity prediction task. It shows the molecules of the neurons where the VX-680 and the pha-739358 are located.

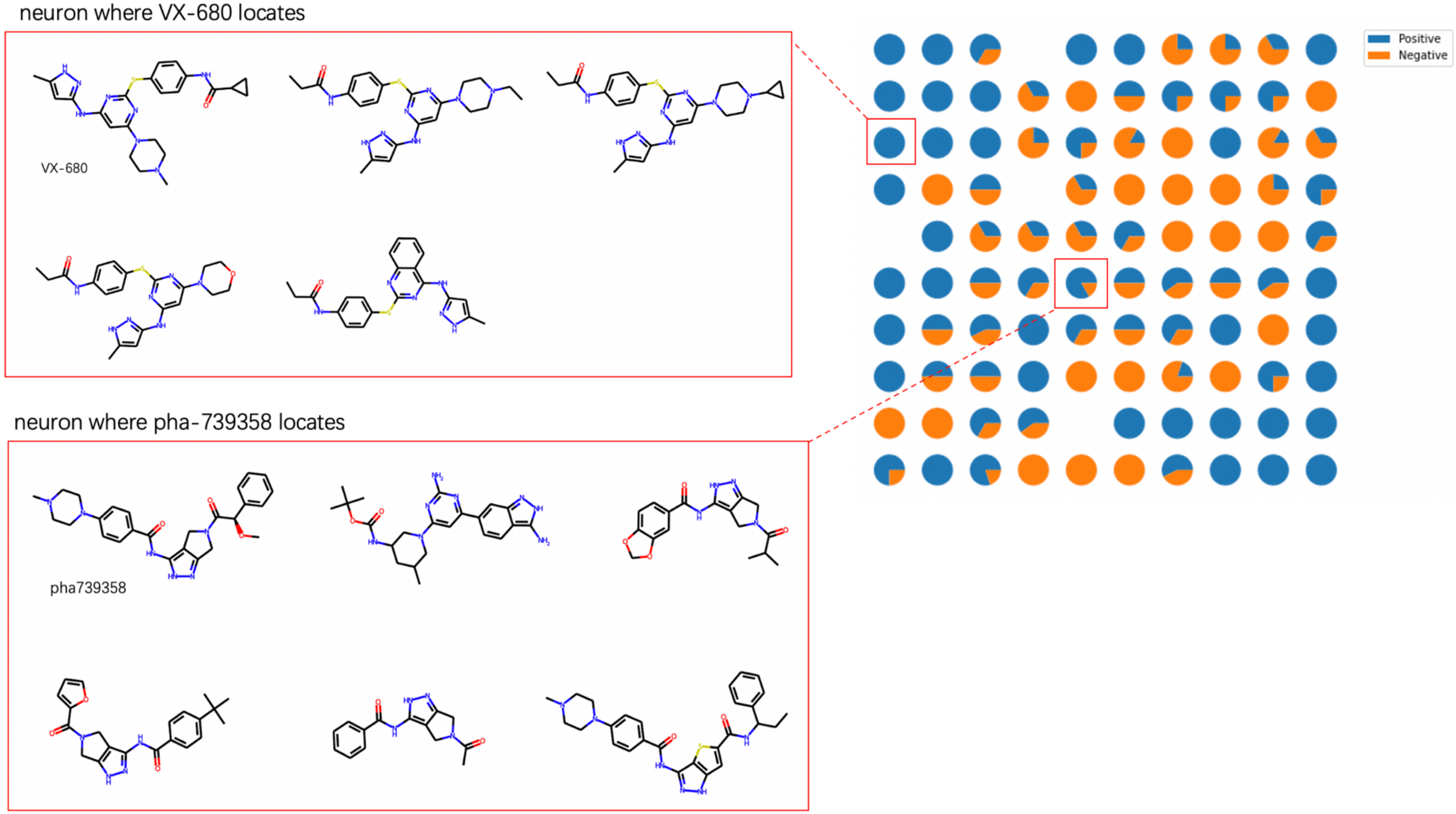

Figure S3. The SOM of the ECFP_4 fingerprints for the AURKA inhibitors. It shows the molecules of the neurons where the VX-680 and the pha-739358 are located.

Reference

S1. Wang M, Xuan S, Yan A, Yu C (2015) Classification Models of HCV NS3 Protease Inhibitors Based on Support Vector Machine (SVM). CCHTS 18:24–32. https://doi.org/10.2174/1386207317666141120122554

S2. Zhang S, Li Y, Qin Z, et al (2019) SAR study on inhibitors of GIIA secreted phospholipase A 2 using machine learning methods. Chem Biol Drug Des 93:666–684. https://doi.org/10.1111/cbdd.13470

S3. Li Y, Tian Y, Qin Z, Yan A (2018) Classification of HIV-1 Protease Inhibitors by Machine Learning Methods. ACS Omega 3:15837–15849. https://doi.org/10.1021/acsomega.8b01843

S4. Wu Y, Huo D, Chen G, Yan A (2021) SAR and QSAR research on tyrosinase inhibitors using machine learning methods. SAR and QSAR in Environmental Research 32:85–110. https://doi.org/10.1080/1062936X.2020.1862297

S5. Wollenhaupt S, Baumann K (2014) inSARa: intuitive and interactive SAR interpretation by reduced graphs and hierarchical MCS-based network navigation. J Chem Inf Model 54:1578–1595. https://doi.org/10.1021/ci4007547

S6. Xiong Z, Wang D, Liu X, et al (2020) Pushing the Boundaries of Molecular Representation for Drug Discovery with the Graph Attention Mechanism. J Med Chem 63:8749–8760. https://doi.org/10.1021/acs.jmedchem.9b00959

S7. Wu Z, Ramsundar B, Feinberg EN, et al (2018) MoleculeNet: a benchmark for molecular machine learning. Chem Sci 9:513–530. https://doi.org/10.1039/C7SC02664A

S8. Yang K, Swanson K, Jin W, et al (2019) Analyzing Learned Molecular Representations for Property Prediction. J Chem Inf Model 59:3370–3388. https://doi.org/10.1021/acs.jcim.9b00237

S9. Jiang D, Wu Z, Hsieh C-Y, et al (2021) Could graph neural networks learn better molecular representation for drug discovery? A comparison study of descriptor-based and graph-based models. J Cheminform 13:12. https://doi.org/10.1186/s13321-020-00479-8
